# B cells require DOCK8 to elicit and integrate T cell help when antigen is limiting

**DOI:** 10.1126/sciimmunol.add4874

**Published:** 2024-08-09

**Authors:** Mukta Deobagkar-Lele, Greg Crawford, Tanya L. Crockford, Jennifer Back, Rose Hodgson, Aneesha Bhandari, Katherine R Bull, Richard J. Cornall

**Affiliations:** 1 MRC Human Immunology Unit, Weatherall Institute of Molecular Medicine, Nuffield Department of Medicine, University of Oxford, Oxford; 2 Centre for Human Genetics, Nuffield Department of Medicine, University of Oxford, Oxford; 3 CAMS-Oxford Institute, Nuffield Department of Medicine, University of Oxford, Oxford; 4 Oxford Kidney Unit, Oxford University Hospitals Trust, Oxford

## Abstract

Dedicator of cytokinesis 8 (DOCK8) immunodeficiency syndrome is characterized by a failure of the germinal center response, a process involving the proliferation and positive selection of antigen-specific B cells. Here, we describe how DOCK8-deficient B cells are blocked at a light-zone checkpoint in the germinal centers of immunized mice, where they are unable to respond to T cell–dependent survival and selection signals, and consequently differentiate into plasma cells or memory B cells. Although DOCK8-deficient B cells can acquire and present antigen to initiate activation of cognate T cells, integrin upregulation, B–T cell conjugate formation, and costimulation are insufficient for sustained B and T cell activation when antigen availability is limited. Our findings provide an explanation for the failure of the humoral response in DOCK8 immunodeficiency syndrome and insight into how the level of available antigen modulates B–T cell cross-talk to fine-tune humoral immune responses and immunological memory.

## Introduction

The germinal center (GC) is a transient and complex microstructure in secondary lymphoid organs and an important site for the development of an effective humoral response. However, the factors that underpin GC initiation, maintenance and dissolution are incompletely understood. In humans, loss-of-function mutations in dedicator of cytokinesis 8 (*DOCK8*) result in a rare, autosomal recessive primary immunodeficiency, with recurrent infections, higher prevalence of allergies and cancers, elevated IgE, and a lack of long-term immune memory (1). Although multiple pathogenic mechanisms contribute to the complex disease phenotype, the inability of DOCK8-deficient B cells to persist in GCs can explain the absence of humoral memory (2–6). Understanding how DOCK8 sustains GC B cells can provide insights into how robust immune responses are generated and maintained in health and disease.

An effective GC reaction requires the coordinated recruitment, selection, and differentiation of B cells and is exquisitely sensitive to any perturbations within this multimodal complex. Upon antigen encounter, naïve B cells are recruited into the GC, where they undergo multiple cycles of proliferation, receptor editing, and somatic hypermutation in the dark zone (DZ). They then compete for antigen and for selection signals from follicular dendritic cells (FDCs) and follicular helper T (T_FH_) cells within the light zone (LZ) (7–10). Although impaired signaling downstream of B cell receptor (BCR) and Toll-like receptor (TLR) engagement have been reported in DOCK8 deficiency (11, 12), the reason for the loss of antigen-activated GC B cells is not known.

Competition for T cell help within the GC is a dynamic process with multiple short and long-lasting GC B–T_FH_ cell interactions that determine survival and affinity maturation (13–15). Integrins and SLAM (signaling lymphocyte activation molecule) family receptors mediate antigen acquisition and cognate B–T cell interactions within the GC (16–18). Although B cell recruitment into the GC and immunoglobulin class switching are intact, DOCK8 modulates the affinity maturation of GC B cells and is required for the recruitment of LFA1–ICAM1 to the BCR immune synapse (2). However, it is unclear whether the loss of DOCK8 compromises the ability of GC B cells to collect antigen from FDCs and interact with T_FH_ cells in the LZ, processes that are critical for GC B cells to avoid deletion from the repertoire and to integrate survival signals for selection into plasma cell (PC) or memory B cell pools, or for re-entry, survival, and proliferation in the DZ (19–23).

To address these questions, we examined the role of DOCK8 within the GC and in naïve primary B cells using the *N*-ethyl-*N*-nitrosourea (ENU)–mutant DOCK8-null *Dock8*^cpm/cpm^ mice (2, 3, 5). We found that B cell–intrinsic DOCK8 sustains the mutual activation of B and T cells when antigen is limited, by fine-tuning the availability of and response to T cell help. These findings have implications for our understanding of how low levels of antigen in the GC create conditions for the selection and affinity maturation of antigen-specific B cells.

## Results

### BCL2 partially rescues DOCK8-deficient germinal center B cells

Studying the role of DOCK8 in GC function is complicated by the almost complete absence of DOCK8-deficient GC B cells. To test for a survival defect in an antigen-specific manner, we crossed the *Dock8*^cpm/cpm^ MD4 mice that carry the anti-hen egg lysozyme (HEL) Ig transgene (2, 24), with mice expressing the B cell–restricted BCL2 transgene (BCL2.Tg) (25).

**Fig. 1:**
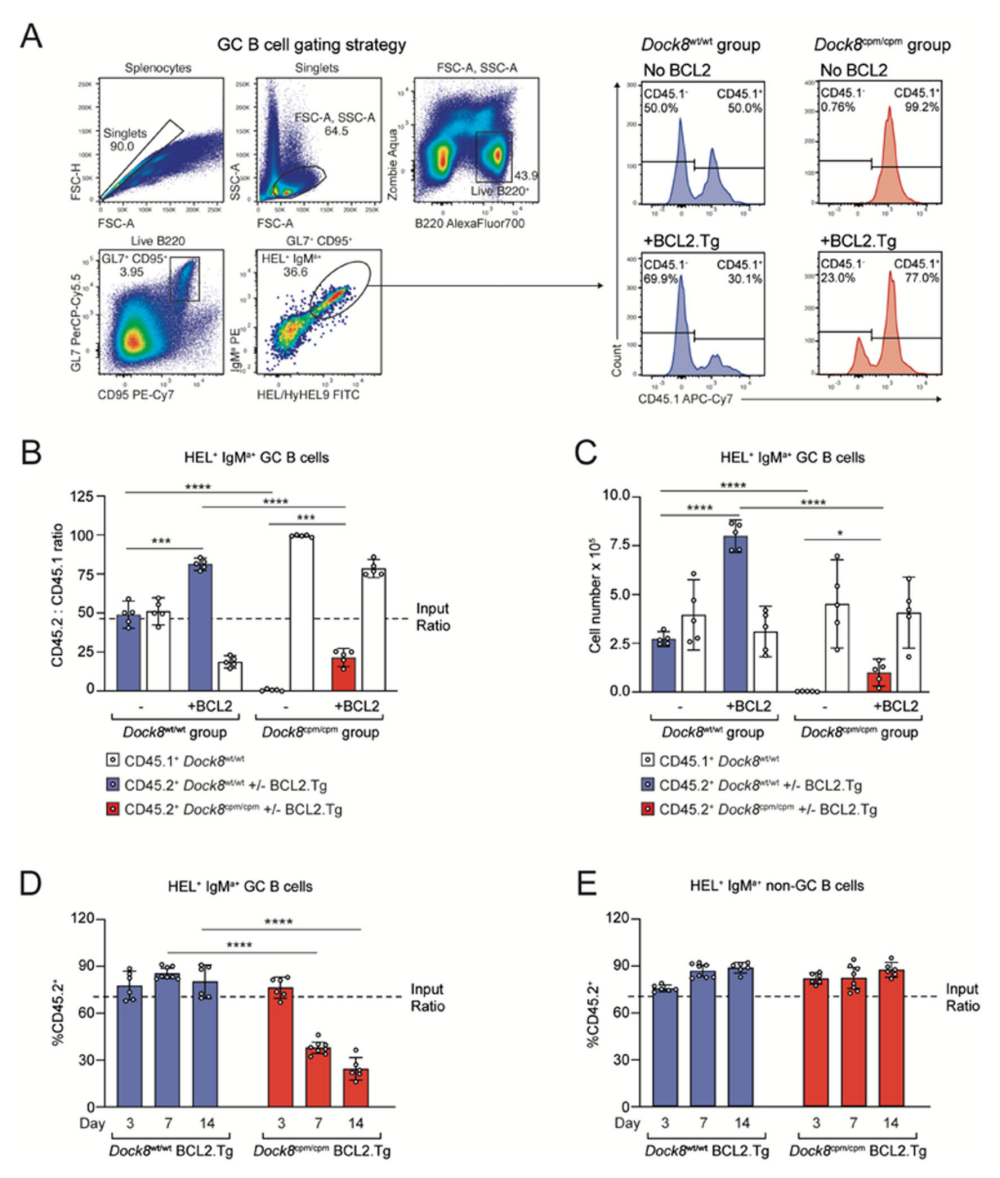
BCL2 partially rescues the loss of DOCK8-deficient GC B cells. CD45.2^+^ *Dock8*^wt/wt^ or *Dock8*^cpm/cpm^ MD4 splenic B cells, with or without BCL2.Tg, were mixed 50:50 with CD45.1^+^ *Dock8*^wt/wt^ MD4 splenic B cells and adoptively transferred into CD45.1^+^ *Dock8*^wt/wt^ C57BL/6 recipients 1 day prior to immunization with SRBC-HEL. Splenic GC populations were analyzed on day 8. (**A**) Gating strategy to identify live CD95^+^GL7^+^IgM^a+^ HEL/HyHEL9^+^(HEL^+^) MD4 splenic GC B cells (left) and representative histograms showing the contribution of CD45.1^+^ and CD45.2^+^ MD4 B cells to the GC. (**B**) Ratios and (**C**) numbers of splenic HEL^+^ GC B cells recovered from indicated groups on day 8 post-immunization. (A to C) Data are representative of three independent experiments, each with *n* = 5 mice per group. (**D** and **E**) CD45.1^+^ *Dock8*^wt/wt^ mice received 70:30 mixtures of CD45.2^+^ BCL2.Tg *Dock8*^wt/wt^ or *Dock8*^cpm/cpm^ MD4 splenic B cells with CD45.1^+^ *Dock8*^wt/wt^ MD4 splenic B cells, 1 day prior to immunization with SRBC-HEL. Ratio of HEL^+^IgM^a+^ splenic (D) GC and (E) non-GC B cells at indicated days post-immunization. Data are pooled from two independent experiments, each with *n* = 3–4 mice per group. (B to E) Circles represent individual mice and bars show means with 95% confidence intervals. Statistics are reported as *P<0.05, ***P<0.001, and ****P<0.0001 by unpaired two-tailed Welch’s *t* test.

Following adoptive transfer of CD45.1^+^ and CD45.2^+^ B cell mixtures, and immunization with sheep red blood cells conjugated to HEL (SRBC-HEL), CD45.2^+^ *Dock8*^cpm/cpm^ MD4 B cells were almost entirely absent from the GC on day 8 (Fig. 1, A to C). CD45.1^+^ and CD45.2^+^ *Dock8*^wt/wt^ MD4 B cells contributed to the GC in the same ratio as input (Fig. 1B). By contrast, BCL2.Tg CD45.2^+^ *Dock8*^wt/wt^ GC B cells had a significant competitive advantage, although BCL2 only partially rescued CD45.2^+^ *Dock8*^cpm/cpm^ GC B cells (Fig. 1, B and C). To assess the persistence of DOCK8-deficient B cells, we analyzed their status both within and outside the GC on days 3, 7, and 14 post-immunization. Although DOCK8-deficient BCL2.Tg MD4 B cells survived in the non-GC B cell compartment over this period and were recruited into the early GC by day 3, they did not persist in the GC over time (Fig. 1, D and E). We confirmed the predominantly GC localization of *Dock8*^wt/wt^ and *Dock8*^cpm/cpm^ BCL2.Tg GFP^+^ MD4 B cells by histological analysis of spleen samples from immunized mice on day 8 (fig. S3, C and D). Thus, DOCK8-deficient B cells require both BCL2-dependent and BCL2-independent signals for survival in the GC.

### DOCK8-deficient germinal center B cells accumulate at a light-zone checkpoint

We harnessed the partial rescue of BCL2.Tg *Dock8*^cpm/cpm^ GC B cells to investigate the B cell–intrinsic role of DOCK8 within the GC by sequencing hashtag-labeled HEL/HyHEL9^+^(HEL^+^)CD95^+^CD45.1^+^ *Dock8*^wt/wt^ (WT) and HEL^+^CD95^+^CD45.2^+^ BCL2.Tg *Dock8*^wt/wt^ or *Dock8*^cpm/cpm^ MD4 GC B cells on a 10X scRNASeq platform (fig. S1A).

The integrated and filtered single-cell dataset consisted of 15,917 GC B cells with distinct G1 and S/G2M cell-cycle phases (fig. S1B). These were consistent with the predominantly resting and proliferative states of cells with LZ and DZ signatures, respectively (fig. S1D). Using classical GC B cell markers and comparing differentially expressed genes (DEGs) with published data sets, we identified six clusters: T cells, PCs, and DZ, LZ_1, LZ_2, and early GC/activated/memory-like (E/A/M) B cells (fig. S1, E to G). Pseudotime analysis suggested two trajectories: first from the E/A/M cluster through the LZ to DZ and back to the LZ cluster, and second from the E/A/M cluster to the LZ and PC clusters. (fig. S1, C and E).

**Fig. 2:**
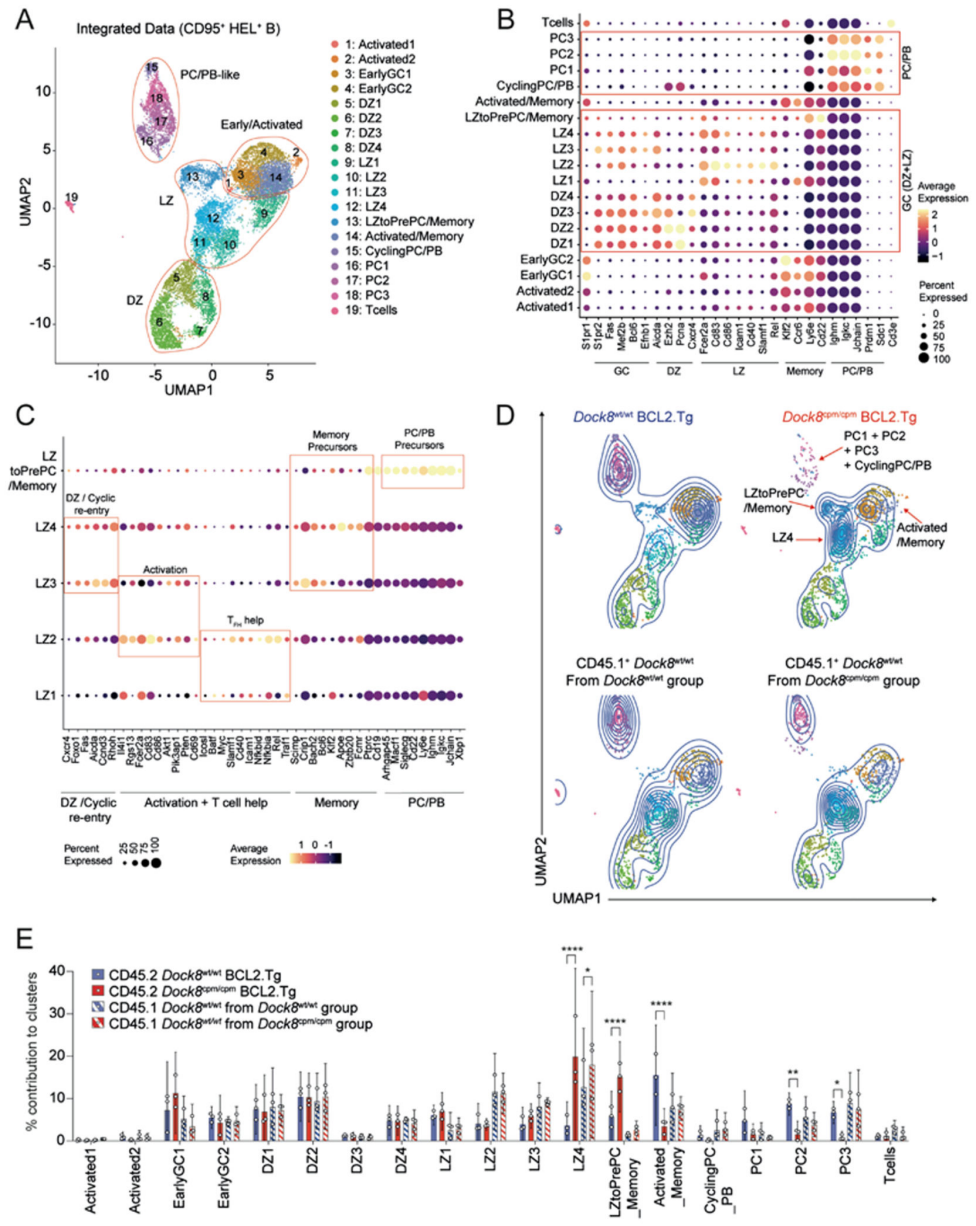
DOCK8-deficient GC B cells accumulate in late LZ clusters enriched for early PC/Memory cell signatures. (**A**) UMAP for the integrated dataset of HEL^+^IgM^a+^CD95^+^ splenic MD4 B cells from six CD45.1 *Dock8*^wt/wt^ samples, three CD45.2^+^ BCL2.Tg *Dock8*^cpm/cpm^ samples, and three CD45.2^+^ BCL2.Tg *Dock8*^wt/wt^ samples showing 19 clusters at resolution 0.9. Clusters are numbered and cells are colored according to cluster identity. (**B**) Differential expression of indicated marker genes across 19 clusters in the integrated dataset. (**C**) DEG between the five LZ clusters. (**D**) UMAP contour plots of the CD45.2^+^ BCL2.Tg subset (upper panel, *Dock8*^wt/wt^ or *Dock8*^cpm/cpm^ samples) and the CD45.1^+^ subset (lower panel, *Dock8*^wt/wt^ samples from the *Dock8*^wt/wt^ and the *Dock8*^cpm/cpm^ groups) with the 19 clusters colored as in (A). (**E**) Distribution of CD45.2^+^ (*Dock8*^wt/wt^ and *Dock8*^cpm/cpm^) and CD45.1^+^ (*Dock8*^wt/wt^) B cells across the 19 clusters. Circles represent individual mice (*n* = 3) and bars indicate means with 95% confidence intervals. Data were analyzed using two-way ANOVA followed by Tukey’s multiple-comparison test. Statistics are reported as *P<0.05, **P<0.01, and ****P<0.0001.

Unsupervised clustering at a higher resolution identified 19 unique clusters with distinct transcriptional profiles including four PC/plasmablast (PB), four DZ, and five LZ clusters (Fig. 2, A and B; fig. S1H; and data S1). Few transcriptional differences were detected within each cluster between *Dock8*^wt/wt^ and *Dock8*^cpm/cpm^ groups in the absence of DOCK8 (fig. S2A and data S2). Among the LZ groups, LZ1 was the most like naïve B cells, LZ2 was enriched for markers of activation and T cell help, and LZ3 and LZ4 were distinguished by high expression of DZ and cell-cycle re-entry markers. In addition, memory precursor markers were enriched in LZ3 and LZ4, and PC/PB markers were highest in LZtoPrePC/Memory cluster (Fig. 2, B and C, and fig. S2, B to E).

Control CD45.1^+^ *Dock8*^wt/wt^ MD4 B cells were comparably distributed across the 19 clusters, with a small increase in the contribution of WT cells from the DOCK8-deficient group to LZ4 (Fig. 2, D and E). However, CD45.2^+^ *Dock8*^cpm/cpm^ B cells were underrepresented in the Activated/Memory-like and PC/PB clusters, and they contributed significantly more to the LZ4 cluster, enriched for *Klf2, Apoe, Zbtb20*, and *Fcmr* and to the LZtoPrePC/Memory cluster enriched for *Arhgap45, Macf1, Siglecg, Cd22, Ly6e, Xbp1*, and *Irf4* (Fig. 2, D and E, and data S1). Although DOCK8 deficiency did not alter DZ composition or contribution to early LZ clusters, a subset of LZ GC B cells expressing memory/PC/PB precursor markers accumulated with a corresponding loss from late GC PC/PB and activated/memory-like clusters. Consistent with the accumulation of DOCK8-deficient B cells in specific LZ clusters, the DZ:LZ ratio of DOCK8-deficient GC B cells was slightly lower (fig. S3, A and B). Furthermore, histological analyses suggested that these cells were predominantly located in the LZ of the GC (fig. S3, C and D). Additionally, fewer CD138^+^IRF4^+^B220^lo/−^ PCs (fig. S3E) and IRF4^+^ GC B cells (fig. S3, F and G) were recovered post-immunization from the DOCK8-deficient B cell pool. Thus, DOCK8 is required at a positive selection/commitment checkpoint in the LZ.

### DOCK8 is required for germinal center B cell survival but not for proliferation

**Fig. 3:**
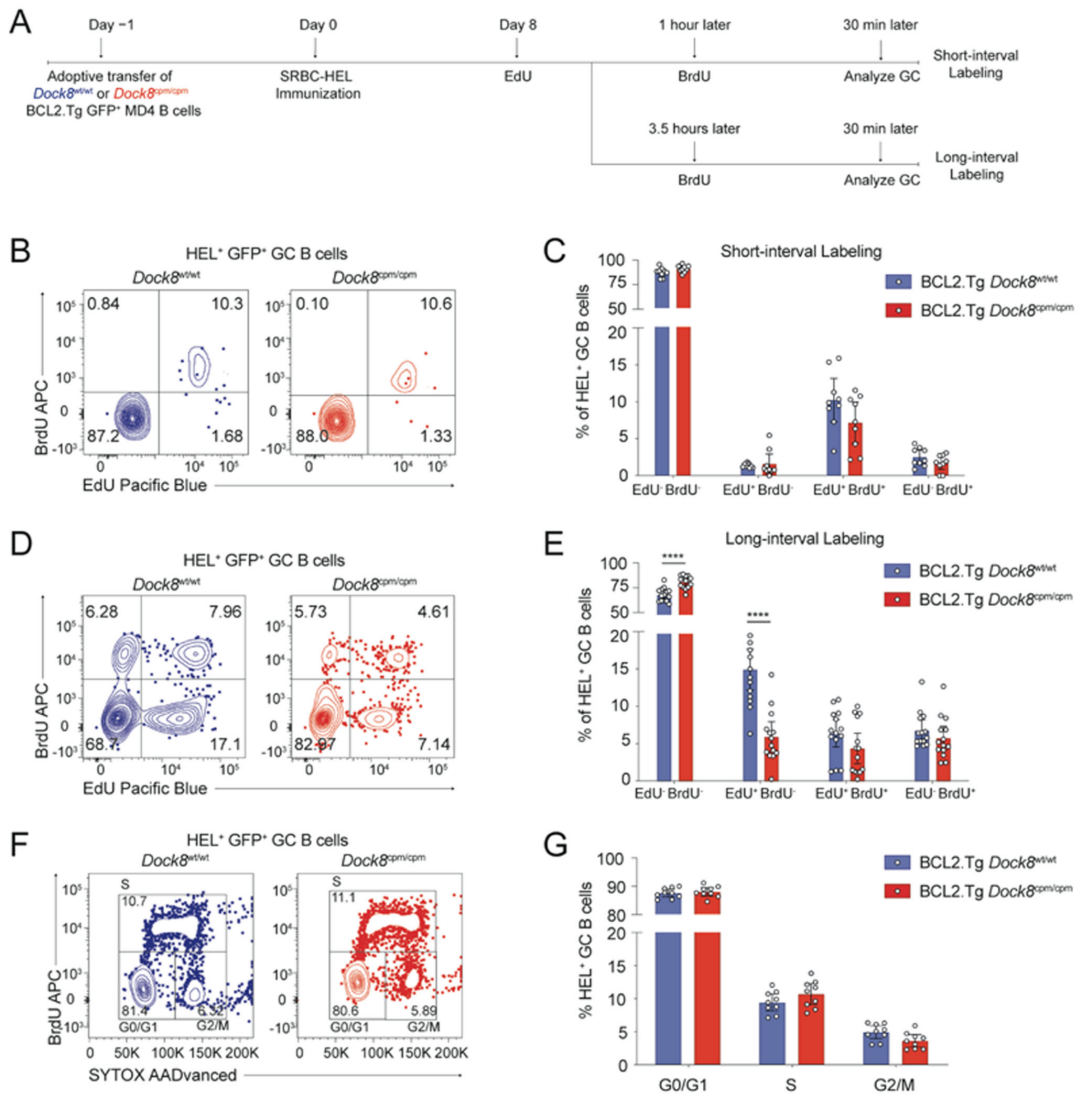
DOCK8 supports GC B cell survival but is not required for cell-cycle progression. (**A**) Schematic for adoptive transfer of *Dock8*^wt/wt^ or *Dock8*^cpm/cpm^ BCL2.Tg GFP^+^ MD4 B cells into C57BL/6 recipients 1 day prior to immunization with SRBC-HEL. On day 8, mice were administered the first nucleotide label (2 mg of EdU), followed by a second nucleotide label (3 mg of BrdU) after an interval of either 1 or 3.5 hours. Splenic B cells were assessed for GC status 30 min after the BrdU dose. (**B** and **D**) Representative contour plots for EdU and BrdU incorporation by MD4 GC B cells (Live B220^+^CD95^+^GL7^+^IgM^a+^GFP^+^ HEL^+^) at (B) 1.5 and (D) 4 hours after the first EdU dose. (**C** and **E**) Proportion of unlabeled, BrdU^+^, EdU^+^, and BrdU^+^EdU^+^ cells from samples as in (B and D), respectively. Data pooled from (C) two and (E) three independent experiments with *n* = 3–6 mice per group. (**F**) Representative contour plots for B220^+^CD95^+^GL7^+^IgM^a+^GFP^+^HEL^+^ B cells in G0/G1, S, and G2/M cell cycle stages. (**G**) Proportion of cells in each cell cycle stage 30 min after BrdU dose with data pooled from two independent experiments with *n* = 4–5 mice per group. (C, E and G) Circles represent individual mice and bars show means with 95% confidence intervals. Unpaired two-tailed Welch’s *t* tests, corrected for multiple comparisons using the Holm–Šídák method were used for analysis with ****P<0.0001.

To determine whether the LZ block and loss of *Dock8*^cpm/cpm^ GC B cells is due to an inability to respond to positive selection signals, we assessed the in vivo proliferation of *Dock8*^wt/wt^ or *Dock8*^cpm/cpm^ BCL2.Tg MD4 GC B cells on day 8 using a dual-nucleotide labeling assay (Fig. 3A) (26). In vivo BrdU uptake by *Dock8*^wt/wt^ or *Dock8*^cpm/cpm^ BCL2.Tg MD4 GC B cells at 30 min was comparable with both the short- and long-interval protocols, indicating that S phase DNA replication is not affected by loss of DOCK8. Furthermore, in the short-interval protocol, DOCK8 did not affect the early (BrdU^+^EdU^−^), mid (BrdU^+^EdU^+^), or late (BrdU^−^EdU^+^) stages of the S phase (Fig. 3, B and C). By contrast, fewer EdU^+^ *Dock8*^cpm/cpm^ GC B cells that had been in S phase 4 hours prior to the endpoint were recovered in the long-interval protocol (Fig. 3, D and E). *Dock8*^wt/wt^ and *Dock8*^cpm/cpm^ B cells had comparable BCL2 expression (fig. S4D) and contributed equivalently to the G0/G1, S and G2/M cell cycle stages in the GC subset (Fig. 3, F and G). Thus, DOCK8 does not appear to be required for DNA synthesis and proliferation but likely supports the survival of GC B cells during post-proliferation selection.

### DOCK8 modulates germinal center B cell activation and adhesion markers

To assess the activation state of DOCK8-deficient GC B cells, we compared the expression of cell surface proteins on GC B cells. Although the expression of GC-identity markers BCL6 and IL21R was equivalent (fig. S4A), the expression of activation markers CD69 and CD86 and adhesion molecules ICAM1, ICAM2, and SLAMF1 was reduced on *Dock8*^cpm/cpm^ GC B cells (fig. S4, B and C). These data led us to hypothesize that DOCK8-deficient B cells may be less able to acquire sufficient T cell help.

### B cells require DOCK8 to acquire T cell help in the germinal center

**Fig. 4:**
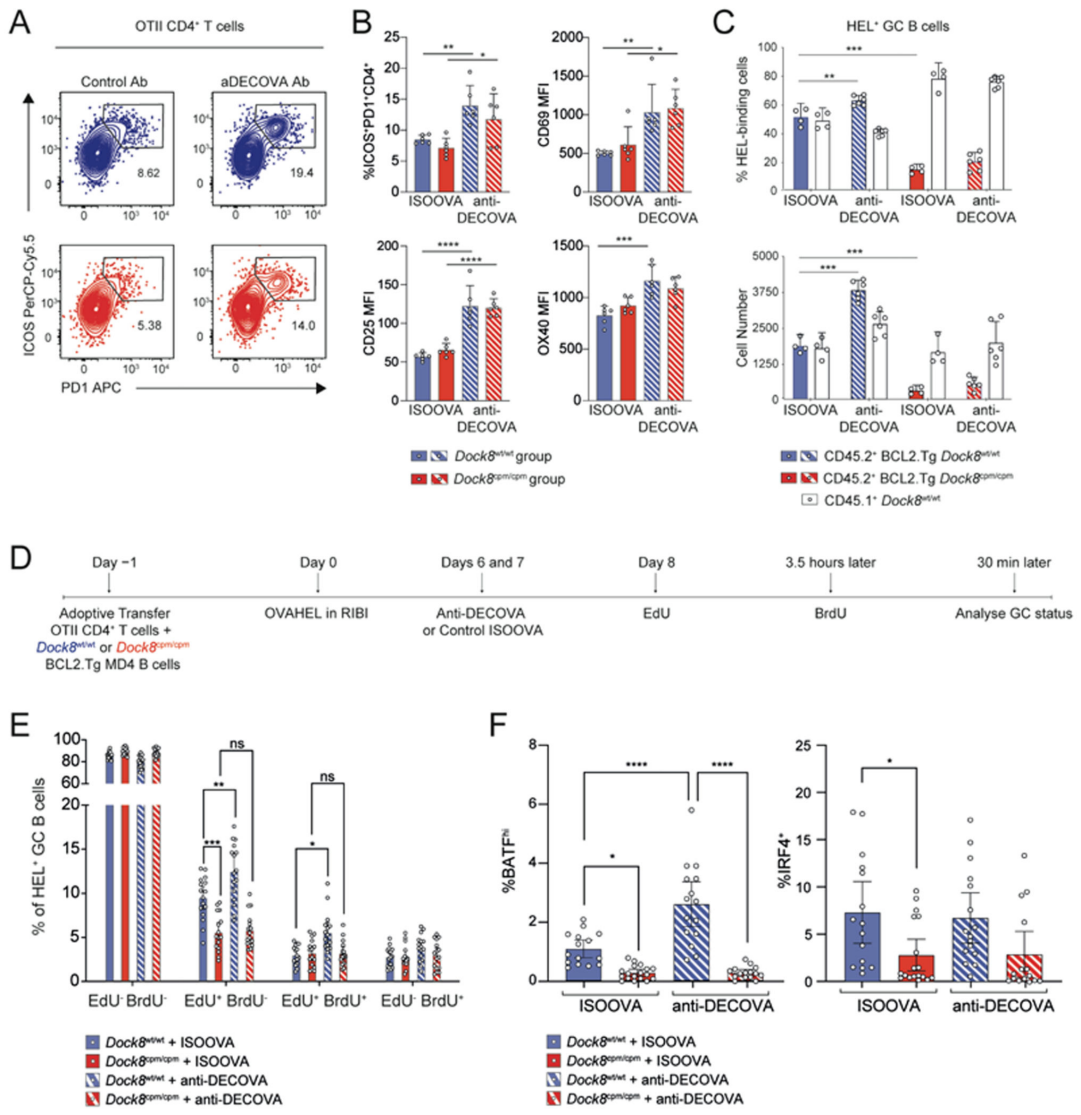
DOCK8-deficient GC B cells are deficient in acquiring T cell help. (**A** to **C**) CD45.2^+^ *Dock8*^wt/wt^ or *Dock8*^cpm/cpm^ BCL2.Tg GFP^+^ MD4 splenic B cells were mixed 50:50 with CD45.1^+^ *Dock8*^wt/wt^ MD4 splenic B cells, and transferred with WT OTII CD4 T cells into CD45.1^+^ *Dock8*^wt/wt^ C57BL/6 recipients one day prior to immunization with OVAHEL antigen. Either ISOOVA or anti-DECOVA antibodies were administered on days 6 and 7 post-immunization, and splenic GC status was evaluated on day 8. (A) Representative contour plots for WT TCRVα2^+^ CD45.2^+^CD4^+^ OTII splenic T cells from *Dock8*^wt/wt^ (top, in blue) or *Dock8*^cpm/cpm^ (bottom, in red). (B) Proportion of ICOS^+^PD1^+^CD4^+^ T_FH_ cells with their activation status. Data are representative of two independent experiments with 4–6 mice per group. (C) Ratios (top) and cell numbers (bottom) of CD45.1^+^ *Dock8*^wt/wt^ and CD45.2^+^GFP^+^ BCL2.Tg *Dock8*^wt/wt^ or *Dock8*^cpm/cpm^ HEL^+^ GC B cells from ISOOVA and anti-DECOVA groups. Data are representative of four independent experiments with *n* = 4–6 mice per group. (**D**) Schematic for adoptive transfer of *Dock8*^wt/wt^ or *Dock8*^cpm/cpm^ BCL2.Tg GFP^+^ MD4 splenic B cells mixed with WT OTII CD4 T cells one day prior to immunization with OVAHEL. Mice received antibody treatment and EdU/BrdU at indicated time points before analysis of splenic GC status. (**E**) Proportion of EdU- and/or BrdU-labeled and (**F**) proportion of BATF^hi^ or IRF4^+^CD45.2^+^ HEL^+^ GC B cells from mice treated as in (D). Data are pooled from three independent experiments with *n* = 4–7 mice per group. (B, C, E, and F) Symbols indicate individual mice and error bars indicate 95% confidence intervals. Statistical analyses using ANOVA with Tukey’s or Šídák’s multiple-comparison test are reported with *P<0.05, **P<0.01, ***P<0.001, and ****P<0.0001.

Since T cell help in the GC is proportional to the amount of peptide–MHC (pMHC) presented (15, 27) and since GC B cells, specifically LZ centrocytes, upregulate expression of the scavenger receptor DEC205 (9, 15, 28, 29), we used an ovalbumin (OVA)-conjugated anti-DEC205 antibody (anti-DECOVA) to evaluate the role of B cell–intrinsic DOCK8 in antigen capture and presentation to T_FH_ cells (30). More ICOS^+^PD1^+^CD4^+^ T_FH_ cells with comparable upregulation of CD69, CD25 and OX40 were recovered from both the *Dock8*^wt/wt^ and *Dock8*^cpm/cpm^ groups upon anti-DECOVA administration, suggesting that the targeted antigen elicited additional T cell help (Fig. 4, A and B). However, although the number of *Dock8*^wt/wt^ BCL2.Tg MD4 GC B cells increased twofold with anti-DECOVA, the contribution of *Dock8*^cpm/cpm^ BCL2.Tg B cells to the GC was unaltered (Fig. 4C). Thus, the GC B cell response remains compromised even when extra T cell help is available, suggesting that deficient antigen capture and presentation via the BCR is unlikely to be the primary cause of GC B cell loss in the absence of DOCK8.

Since T_FH_ cell help accelerates GC B cell-cycle progression (26), we repeated our analysis of the proliferation and survival of GC B cells using dual EdU–BrdU long-interval labeling in the context of anti-DECOVA (Fig. 4D). Despite the increased recovery of EdU-labeled *Dock8*^wt/wt^ BCL2.Tg GC B cells (both EdU^+^BrdU^−^ and EdU^+^BrdU^+^) from the anti-DECOVA–treated group, a comparable increase was not observed in the absence of DOCK8 (Fig. 4E). BATF is upregulated by CD40 signaling in GC LZ B cells that have recently received T cell help (31, 32). The DOCK8-deficient GC pool had significantly fewer BATF^+^IRF4^−^ and BATF^+^IRF4^+^ (BATF^hi^) and BATF^lo/−^IRF4^hi^ (IRF4^+^) cells (Fig. 4F and fig. S3, F and G). Anti-DEC205 increased the proportion of BATF^hi^ *Dock8*^wt/wt^ GC B cells, whereas DOCK8-deficient GC B cells were unaffected (Fig. 4F). Thus, B cells require DOCK8 to receive and respond to T cell help in the GC.

### DOCK8 is required to elicit T cell help when antigen is limiting

To dissect the role of DOCK8 in B and T cell interactions, we measured the in vivo proliferation of *Dock8*^wt/wt^ or *Dock8*^cpm/cpm^ MD4 B cells, that were loaded with OVA–HEL (OVAHEL) antigen ex vivo and mixed with cognate WT OTII CD4 T cells before adoptive transfer. After 72 hours, OTII CD4 T and MD4 B cells in the *Dock8*^wt/wt^ group had proliferated in an antigen dose–dependent manner. However, the proliferation of both OTII CD4 T cells and MD4 B cells from the *Dock8*^cpm/cpm^ group was compromised at HEL antigen amounts of 500 ng/ml or lower (fig. S5A), indicating that DOCK8 is required for mutual activation of B and CD4 T cells at these doses.

**Fig. 5:**
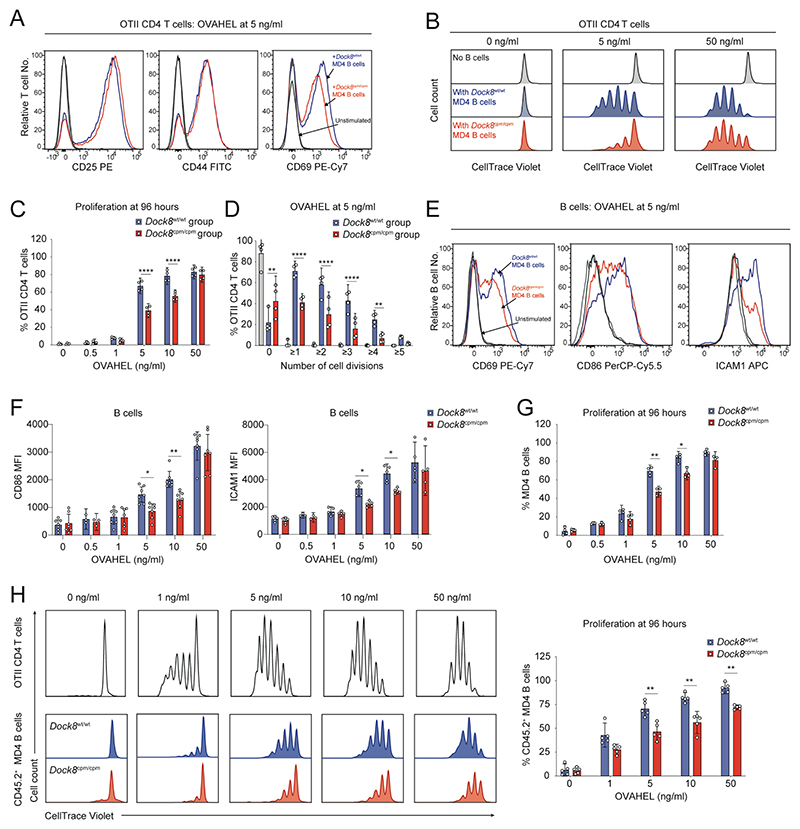
DOCK8 is required for B and T cell activation when antigen is limiting. (**A**) Representative histograms of activation markers at 16 hours and (**B**) of proliferation at 96 hours for WT OTII CD4 T cells cultured with *Dock8*^wt/wt^ or *Dock8*^cpm/cpm^ MD4 splenic B cells at indicated levels of OVAHEL. (**C**) Proliferation of WT OTII CD4 T cells at indicated levels of OVAHEL and (**D**) proportion of T cells that have undergone indicated number of cell divisions with 5 ng/ml of OVAHEL at 96 hours. (**E**) Representative histograms and (**F**) expression levels of activation markers at 16 hours, and (**G**) proliferation at 96 hours of *Dock8*^wt/wt^ or *Dock8*^cpm/cpm^ MD4 splenic B cells cultured with WT OTII CD4 T cells at indicated levels of OVAHEL. Data in (A, B and E) are representative of four independent experiments. Data in (C, D, F, and G) are pooled from four independent experiments. (**H**) Representative histograms (left) and pooled data (right) for proliferation of OTII CD4 T cells and CD45.2^+^ MD4 B cells at 96 hours in mixed cultures of WT OTII CD4 T cells, CD45.1^+^ *Dock8*^wt/wt^ and CD45.2^+^ *Dock8*^wt/wt^ or *Dock8*^cpm/cpm^ MD4 splenic B cells at indicated levels of OVAHEL. Data are pooled from five independent experiments. Bars show means, symbols indicate data from individual experiments and error bars indicate 95% confidence intervals. Data were analyzed by two-way ANOVA with Tukey’s multiple-comparison test (C and D) and by unpaired Welch’s *t* tests corrected for multiple comparisons using the Holm–Šídák method (F to H) with *P<0.05, **P<0.01, and ****P <0.0001.

To investigate whether there was a general defect in the response of DOCK8-deficient B cells to low levels of antigen, we cultured MD4 B cells and WT OTII CD4 T cells with OVAHEL ex vivo (fig. S5B). Although OTII CD4 T cells expressed comparable levels of the activation markers CD25, CD44, and CD69 after 16 hours (Fig. 5A and fig. S5C), fewer OTII CD4 T cells cultured with *Dock8*^cpm/cpm^ MD4 B cells proliferated at 5 and 10 ng/ml of OVAHEL, levels that are not saturating for the MD4 BCR (Fig. 5, B to D, and fig. S5D). Although B cell CD69 expression was equivalent after 16 hours (fig. S5E), CD86 and ICAM1 expression was lower on *Dock8*^cpm/cpm^ B cells at 5 and 10 ng/ml (Fig. 5, E and F). *Dock8*^cpm/cpm^ MD4 B cells proliferated less at the lower antigen doses (Fig. 5G and fig. S5F), confirming a general defect in B and T cell activation when antigen is limiting in the absence of DOCK8.

To determine whether the compromised proliferation of *Dock8*^cpm/cpm^ B cells in the presence of cognate T cells and low levels of antigen was intrinsic, we cocultured WT OTII CD4 T cells and CD45.1^+^ *Dock8*^wt/wt^ MD4 B cells with CD45.2^+^ *Dock8*^wt/wt^ or *Dock8*^cpm/cpm^ MD4 B cells and OVAHEL. Although OTII CD4 T cells and *Dock8*^wt/wt^ MD4 B cells proliferated extensively by day 4, fewer CD45.2^+^ *Dock8*^cpm/cpm^ MD4 B cell proliferated at each dose (Fig. 5H), confirming that B cell–intrinsic DOCK8 is required to generate a normal response to T cell help.

### Defective costimulation during B–T cell crosstalk in DOCK8-deficiency

**Fig. 6:**
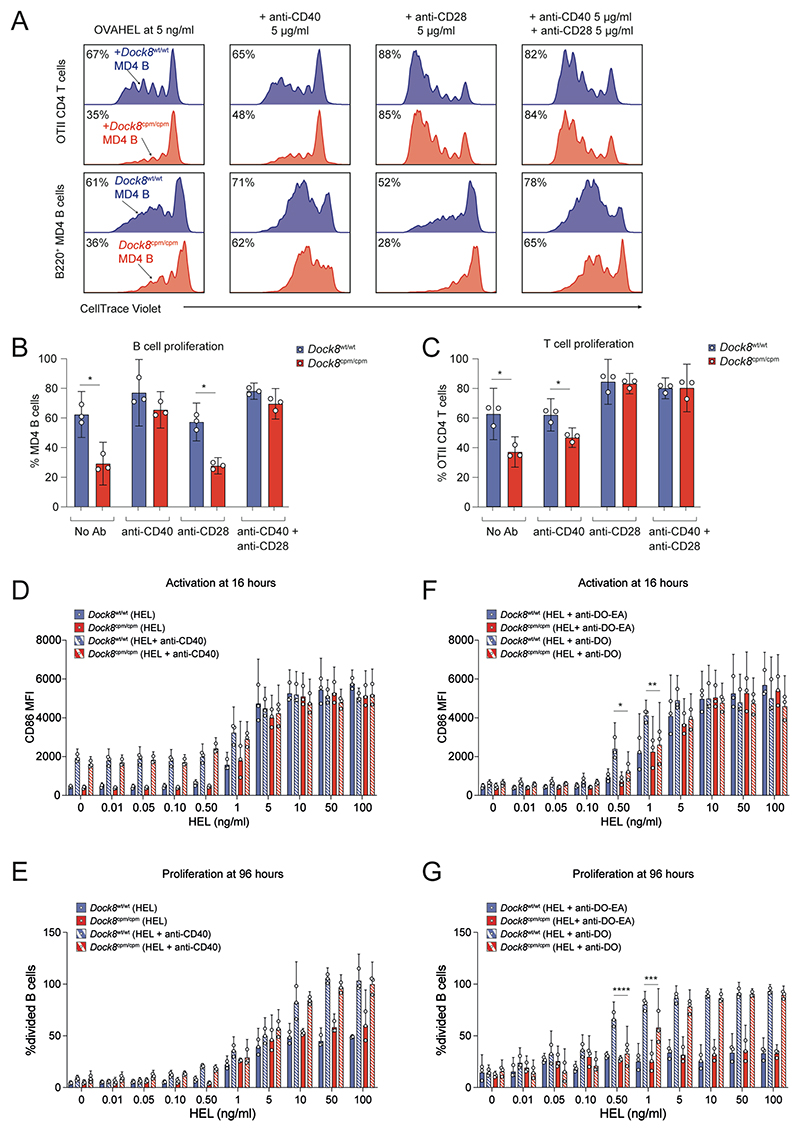
B cells require DOCK8 to integrate costimulatory signals and T cell help. (**A** to **C**) Mixtures of CD45.1^+^ *Dock8*^wt/wt^ (blue) and CD45.2^+^ *Dock8*^cpm/cpm^ (red) MD4 splenic B cells with WT OTII CD4 T cells were labeled with CTV and activated with 5 ng/ml of OVAHEL. (A) Proliferation of OTII CD4 T cells (upper panel) and MD4 B cells (lower panel) was assayed by CTV dilution at 96 hours in the presence of 5 μg/ml each of anti-CD40 or anti-CD28 or a combination of both. Numbers indicate proportion of cells that have proliferated, rounded to nearest integer. Pooled proliferation data for (B) MD4 B cells and (C) OTII CD4 T cells at 96 hours. Cells from two mice per group were used for each experiment and data are pooled from three independent experiments. Data were analyzed by unpaired *t* test with Welch’s correction, and with *P<0.05. (**D** to **G**) CTV-labeled mixtures of *Dock8*^wt/wt^ (blue) or *Dock8*^cpm/cpm^ (red) MD4 splenic B cells with OTII CD4 T cells were cultured with indicated HEL amounts. Pooled data for (D) CD86 expression and (E) proliferation of MD4 B cells with or without 5 μg/ml of anti-CD40. Pooled data for (F) CD86 expression and (G) proliferation of MD4 B cells in the presence of 10 μg/ml each of anti-DECOVA (DO) or anti-DECOVA-EA (DO-EA). Data were analyzed using two-way ANOVA with Šídák’s multiple comparisons test. Statistics are reported as *P<0.05, **P<0.01, ***P<0.001, and ****P <0.0001. (B to G) Cells were pooled from *n* = 2–3 donor mice per group per experiment and data are pooled from three independent experiments. Symbols indicate data from individual experiments, bars show means, and error bars indicate 95% confidence intervals.

Since complete activation of B and T cells requires a signal 1, received upon antigen-engagement of the BCR or TCR, and a pro-survival costimulatory signal 2, we tested whether signal 2 was limiting at low levels of antigen using anti-CD40 or anti-CD28 antibody or a combination of both. The anti-CD40 antibody rescued *Dock8*^cpm/cpm^ MD4 B cell proliferation in a dose-dependent manner but not OTII CD4 T cell proliferation (fig. S6, A and B). By contrast, the anti-CD28 antibody rescued CD4 T cell proliferation in the *Dock8*^cpm/cpm^ group but not the B cell proliferation defect itself (fig. S6, C and D). However, the combination of the two signal 2 surrogates rescued the proliferation of B and CD4 T cells in the *Dock8*^cpm/cpm^ group (Fig. 6, A to C). Thus, DOCK8 is required to maintain costimulatory crosstalk between antigen-presenting B cells and cognate CD4 T cells when the amount of antigen is limiting.

### DOCK8 is dispensable for soluble antigen acquisition and presentation

To assess whether the compromised B–T cell costimulatory interaction is due to defective BCR signaling or antigen uptake, we tested the ability of naïve *Dock8*^wt/wt^ or *Dock8*^cpm/cpm^ MD4 B cells to present OVA antigen, acquired independently of the BCR via DEC205, to WT OTII CD4 T cells (30). Fewer OTII CD4 T cells in the *Dock8*^cpm/cpm^ group proliferated in response to anti-DECOVA compared to those in the *Dock8*^wt/wt^ group, (fig. S7A), suggesting that DOCK8 is required for cognate B–T cell interactions in a manner distinct from BCR-mediated antigen acquisition and signaling.

Next, we investigated the role of DOCK8 in BCR-mediated presentation of soluble antigen and detected comparable levels of Eα(52-68) peptide–bound I-A^b^ MHC class II on *Dock8*^wt/wt^ or *Dock8*^cpm/cpm^ MD4 B cells (33) (fig. S7B). To confirm this observation, we incubated I-A^k^/I-A^b^ *Dock8*^wt/wt^ or *Dock8*^cpm/cpm^ MD4 B cells with HEL, and non-transgenic I-A^k^/I-A^b^ *Dock8*^wt/wt^ or *Dock8*^cpm/cpm^ B cells with anti-DECHEL or control anti-DECOVA, and detected equivalent levels of HEL(46-61) peptide–bound I-A^k^ MHC class II (34) (fig. S7, C and D). Thus, DOCK8 is not required for B cells to collect and present soluble antigen, acquired via the BCR or DEC205, to cognate T cells.

### B cells require DOCK8 to integrate contact-dependent T cell help

Next, we assessed the BCR-independent role of DOCK8 in initiating contact-dependent cognate T cell help. We cultured HEL–stimulated *Dock8*^wt/wt^ or *Dock8*^cpm/cpm^ MD4 B cells with either soluble anti-CD40 antibody—as a surrogate for T cell help—or with OTII CD4 T cells and anti-DECOVA—to elicit BCR-independent but contact-dependent T cell help.

*Dock8*^wt/wt^ or *Dock8*^cpm/cpm^ MD4 B cells were similarly activated by HEL in an antigen dose–dependent manner (Fig. 6, D and F). The surrogate T cell signal via anti-CD40 alone activated MD4 B cells in the absence of HEL and enhanced the HEL dose-dependent proliferation of B cells independently of DOCK8 (Fig. 6, D and E). CD69 was upregulated on WT OTII CD4 T cells at 16 hours by MD4 B cells copresenting OVA antigen acquired via anti-DECOVA in a HEL dose-dependent, but DOCK8-independent, manner (fig. S7E). However, after 96 hours in the presence of anti-DECOVA, and at low HEL concentrations, the OTII CD4 T cells cultured with *Dock8*^cpm/cpm^ MD4 B cells proliferated significantly less than those cultured with *Dock8*^wt/wt^ MD4 B cells (fig. S7F). Furthermore, T cell help elicited via anti-DECOVA enhanced CD86 expression and proliferation of *Dock8*^wt/wt^ MD4 B cells at 0.5 and 1 ng/ml of HEL. However, *Dock8*^cpm/cpm^ MD4 B cells failed to upregulate CD86 and proliferated less at the same low concentrations of HEL in the presence of anti-DECOVA (Fig. 6, F and G). Thus, DOCK8-dependent contact-mediated costimulatory interactions between B and T cells are essential to sustain the mutual activation of B and T cells at low antigen doses, when signaling via BCR engagement is correspondingly low.

### DOCK8 modulates sustained B and T cell contacts

**Fig. 7:**
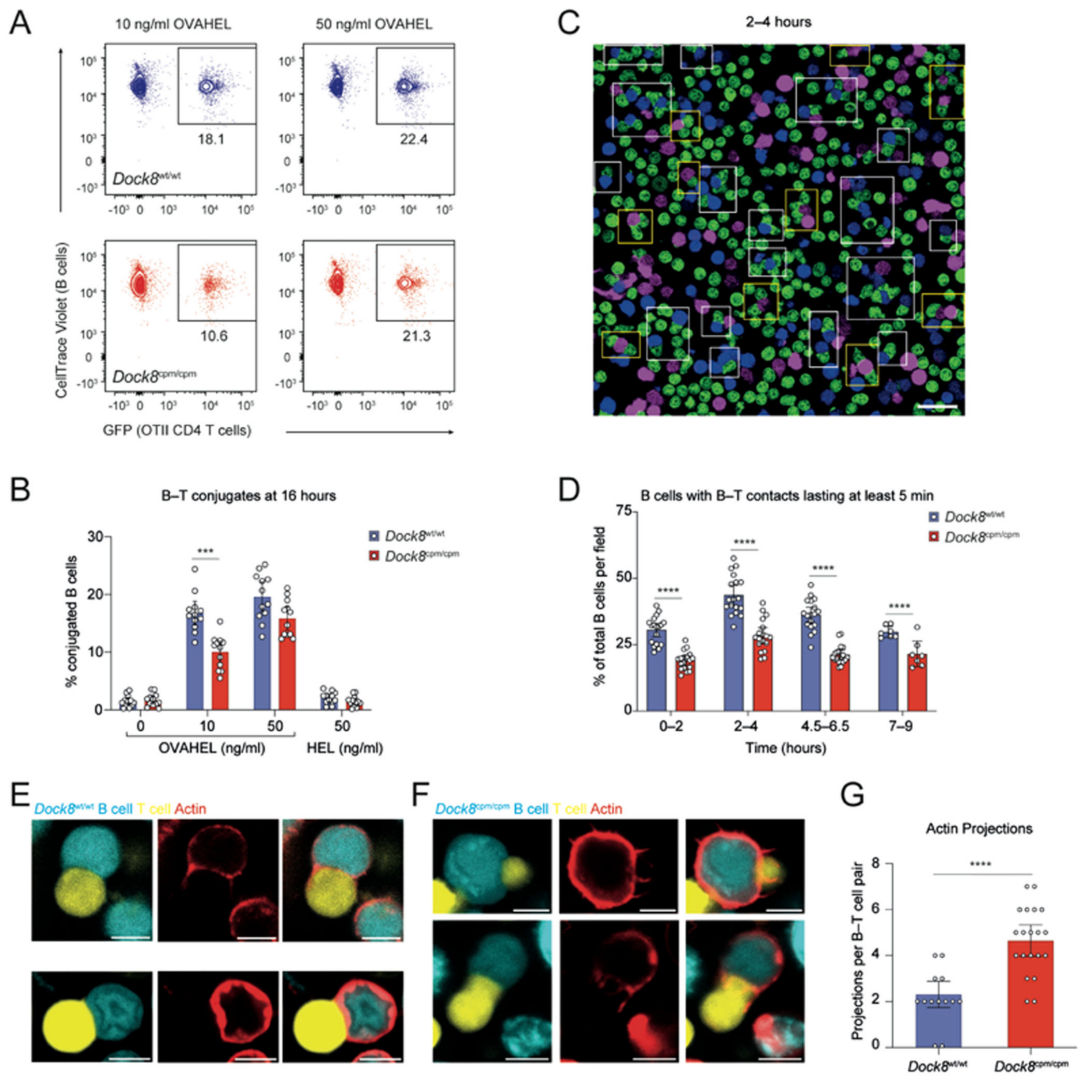
DOCK8 sustains long-lasting B and T cell contacts when antigen is limiting. (**A** and **B**) Purified CTV-labeled *Dock8*^wt/wt^ or *Dock8*^cpm/cpm^ MD4 splenic B cells were cultured with GFP^+^ OTII CD4 T cells and the indicated antigens. B–T cell conjugates quantified at 16 hours. (A) Representative plots and (B) pooled data from three experiments, each with *n* = 4 mice per group. Symbols indicate individual mice. (**C** and **D**) *Dock8*^wt/wt^ (blue) or *Dock8*^cpm/cpm^ (magenta) MD4 splenic B cells, incubated with 10 ng/ml of OVAHEL for 6–12 hours were mixed with OTII CD4 T cells (green). Images were recorded across 7–18 frames for 8–10 hours. (C) Representative image of B–T cell conjugates at 2–4 hours. White boxes indicate T cell conjugates with *Dock8*^wt/wt^ B cells and yellow boxes with *Dock8*^cpm/cpm^ B cells. (D) Proportion of B cells with B–T contacts lasting at least 5 min. Data are representative of three experiments. Cells were pooled from *n* = 2–3 mice per group and symbols indicate proportion of conjugates quantified per frame. (**E** to **G**) CFSE-labeled GC-like B cells (cyan), incubated with 10 ng/ml of OVAHEL for 6–12 hours and labeled with SiR-actin (red), were mixed with CTY-labeled OTII CD4 T cells (yellow) before recording B–T cell conjugates. Representative images for T cell conjugates with (E) *Dock8*^wt/wt^ and (F) *Dock8*^cpm/cpm^ B cells. (G) Data for actin projections at the interface of B–T cell conjugates, pooled from two experiments. Symbols indicate data from 13 *Dock8*^wt/wt^ and 20 *Dock8*^cpm/cpm^ B–T cell conjugates. Scale bars represent 20 μm for (C) and 5 μm for (E and F). For (B, D, and G) bars show means with 95% confidence intervals. Data analyzed by Welch’s *t* test (B and D) and Mann–Whitney test (G), with ***P<0.001 and ****P <0.0001.

Next, we assessed the role of DOCK8 in sustaining B–T cell contacts for conjugate formation and the complete activation of both partners. Significantly more B–T cell conjugates were detected with OVAHEL compared to HEL, confirming that these were cognate interactions (Fig. 7B). However, fewer T cell conjugates with *Dock8*^cpm/cpm^ B cells were detected at 10 ng/ml of OVAHEL antigen (Fig. 7, A and B). We also investigated the formation of conjugates between WT OTII CD4 T cells and antigen-activated *Dock8*^wt/wt^ or *Dock8*^cpm/cpm^ MD4 B cells over time, in the context of limiting antigen and competition. At all time points, *Dock8*^cpm/cpm^ B cells formed fewer conjugates, which were defined as contacts lasting for at least 5 min with a characteristic flattening at the B–T cell contact interface (Fig. 7, C and D; fig. S8, A and B; and movies S1, S2, and S3).

The majority of B–T cell conjugates were 1:1 “monogamous” pairs and their formation was compromised in the absence of DOCK8 at early time points. However, this defect resolved at later time points (fig. S8, A and B). We observed more “polygamous” conjugates consisting of a single B cell and multiple T cells at later time points. However, *Dock8*^cpm/cpm^ B cells formed fewer such polygamous conjugates over time (fig. S8, A and B). By contrast, combinations of one T cell and multiple B cells were less frequently present and were mainly observed in the context of DOCK8-deficient B cells (fig. S8B). Thus, B cells require DOCK8 to form multiple cognate interactions with T cells in order to integrate T cell help within a competitive environment and in the context of low amounts of antigen.

GC-like *Dock8*^cpm/cpm^ B cells were less able to form dendrite-like extensions and spread on anti-CD44 coated surfaces, a process that requires coordinated polymerization of actin and tubulin (35) (fig. S8C), suggesting a role for DOCK8 in cytoskeleton remodeling. To investigate modulation of the B cell cytoskeleton during B–T cell interactions, we imaged the actin cytoskeleton of GC-like B cells using SiR-actin during their cognate interactions with T cells ex vivo. Actin was modulated at the B and T cell contact region with a few small actin protrusions from the B cell at the synapse between *Dock8*^wt/wt^ B cells and cognate T cells (Fig. 7E and fig. S8D). *Dock8*^wt/wt^ B cells formed relatively uniform and stable contacts with T cells (movies S4, S5, S8, and S9), whereas the DOCK8-deficient B cells formed more unstable contacts with significantly more actin protrusions at the synaptic interface (Fig. 7, F and G; fig. S8D; and movies S6, S7, S10, and S11). This unstable interaction at the contact area was suggestive of DOCK8-deficient B cells “slipping” as they attempted to hold onto their T cell partner (fig. S8E and movie S6). Thus, DOCK8 appears to be required for the modulation of actin to maintain stable B and T cell contacts. This is particularly critical when B cells activated with low amounts of antigen compete for contact-mediated T cell help for complete activation (fig. S9).

## Discussion

We have found that the B cell–intrinsic loss of DOCK8 compromises the ability of B cells to interact with T cells and receive and integrate survival signals, which are necessary in competitive environments where antigen is limiting. This is associated with a block in the development of PCs and immune memory and reduced GC B cell survival. Discrete subsets of low- and high-affinity LZ centrocytes, sensitized by interaction with antigen towards a potential PC or memory B cell fate prior to T cell help, require T cell help to complete fate commitment and differentiation (19, 21). Selection into the DZ is cMYC-dependent and cMYC upregulation in the LZ also depends on the extent of T cell help received (23, 36, 37).

Although DOCK8 deficiency does not affect cell cycle entry and the progression of GC B cells through the proliferative phase of the cell cycle, fewer DOCK8-deficient B cells that had previously proliferated were recovered over a 4-hour period. GC B cells are highly susceptible to cell death, and an average of 50% GC B cells are destined to die every 4–6 hours in both the LZ and the DZ unless rescued by positive selection via T_FH_ cells (38–40). Furthermore, DOCK8-deficient GC B cells are not accelerated through the cell cycle in response to extra T_FH_ help, confirming that DOCK8 is required for a B cell–intrinsic ability to receive T cell help. It is likely that the sub-optimal B–T cell interactions result in incomplete “refueling” or recharging of LZ centrocytes such that they complete fewer proliferative cycles during successive DZ periods (31, 41), precipitating a B cell–intrinsic survival defect and compounding the progressive loss of DOCK8-deficient B cells from the GC.

Since DOCK8-deficient B cells can cluster antigen at the BCR synapse but are unable to regulate LFA1–ICAM1 integrin recruitment (2), we cannot rule out a role for DOCK8 in integrin-dependent signals at the FDC and GC B cell contact. We also cannot exclude the possibility that the defects in the B cell synapse that we previously observed may lead to a relative failure to internalize membrane-bound antigen from FDCs. Recent evidence suggests a quantitative and qualitative difference in B cell responses to soluble, membrane-bound and particulate antigens (42). Signaling via the BCR also provides survival signals critical for positive selection in the GC, especially within a competitive setting (43, 44). Defects in antigen gathering may further compound the inability of DOCK8-deficent B cells to receive T cell help, especially in the context of low levels of antigen. However, DOCK8-deficient B cells can present antigen to cognate CD4 T cells and are not rescued by BCR-independent delivery of antigen and T cell help via anti-DECOVA, suggesting that the primary B cell–intrinsic defect underlying the characteristic GC B cell loss in DOCK8 immunodeficiency is downstream of antigen gathering and presentation.

Transient expression of BATF in LZ GC B cells is triggered by T cell–dependent CD40 signaling (32), where it is required for GC maintenance, *Aicda* transcription, and cell cycle progression, with at least some of these functions requiring BATF interactions with IRF4 (32, 45). We have shown that fewer DOCK8-deficient GC B cells have this recent T cell help signature and anti-DECOVA does not increase the proportion of recently helped BATF^hi^ *Dock8*^cpm/cpm^ B cells (31, 32). T cell signals are required for refueling, survival and differentiation of GC B cells and fewer CD138^+^IRF4^+^B220^lo^ DOCK8-deficient PCs are recovered upon immunization. Furthermore, B and T cell conjugate formation and the proliferation of DOCK8-deficient B cells and cognate T cells are compromised ex vivo when antigen amounts are limiting. Thus, our data suggest that the loss of DOCK8-deficient B cells is primarily due to a failure to acquire sufficient T cell help within the GC, at least in part due to a B cell–intrinsic failure to initiate or sustain crosstalk with T_FH_ cells. It is possible that the positive regulation of T cell contacts by DOCK8 may ordinarily allow B cells with a range of antigen-gathering abilities to be retained in the GC, thus allowing for a broad B cell pool and immune response.

B and T cell crosstalk via CD40 and ICOS signaling regulates a positive selection loop in the GC (13, 46), whereas LFA1–ICAM1 interactions lower signaling thresholds and are critical for adhesion and synapse formation (47). ICAM1 and ICAM2 on B cells additively facilitate long-lasting B–T cell contacts, which are important for GC seeding and affinity maturation. Furthermore, interactions between SAP, CD84, and other SLAM family receptors are required for sustained B and T cell conjugates within the GC (14, 16, 17). We found that CD86, ICAM1, and SLAMF1 upregulation was defective in DOCK8-deficient B cells in the GC and in vitro only when the level of antigen was itself limiting, the very situation likely to be required for selection by rare antigens under physiological conditions.

B cell activation requires BCR signaling to prime B cells to receive and integrate second signals via T cell contact or via other receptors. When reduced BCR signaling at low doses of antigen elicits a reduced priming response, DOCK8 facilitates a positive feedback loop by sustaining multiple B–T cell contacts to allow B cells to upregulate costimulatory and adhesion molecules and overcome the signaling threshold. At high or saturating antigen doses that elicit adequate priming, additional input from DOCK8-mediated processes for maintaining long-lasting B–T cell interactions is dispensable. Within the competitive GC environment, ensuring that the B cell response is highly sensitive to the antigen threshold creates conditions in which B cells with relatively higher affinity have a greater chance of being optimally activated (48, 49). Dependence on other signaling pathways, such as those regulated by DOCK8, could allow for the selection of the fittest clones that can not only gather antigen, but can best compete for T cell help, enforcing tolerance while allowing a range of antigenic specificities to persist in the repertoire.

DOCK8 is required for the LFA1/ICAM1–dependent positioning of T_FH_ cells in the GC (50) and inhibits IL-13–driven T_FH_ cell–induced high-affinity IgE production (51), suggesting that DOCK8 supports contact-dependent selection in the GC. Although DOCK8 is necessary for B cells to sustain multiple contacts with T cells over time, we found that bypassing contact dependent T cell help using a surrogate anti-CD40 antibody abolishes the dependence on DOCK8. We found more actin protrusions and ineffective cytoskeletal coordination at the B–T cell interface in the absence of DOCK8, suggesting that DOCK8 regulates cytoskeletal remodeling to stabilize multiple short interactions between cognate B–T cells. Cytoskeletal reorganization may allow the synaptic integration of signals necessary to overcome antigen-dependent activation thresholds (13, 14, 52). The cytoskeleton modulator intersectin 2 (ITSN2) has GEF activity and regulates expression of SLAM, CD84, and ICOSL to promote long-term B–T cell conjugate formation, as well as GC maintenance and memory against vaccination and viral infections (53). Dynamic reorganization of the cytoskeleton facilitates serial and parallel engagements between cognate B and T cells within the GC and elsewhere, and actin cytoskeleton remodeling is important for mechanosensing of signaling thresholds (54, 55). Measurement of forces at the synapse between cognate B–T cell conjugates and the B–T cell transcriptional profiles with antigen thresholds may enhance our understanding of how antigen controls cellular interactions and B cell fate decisions.

The GC B cell loss underlying the humoral defect in DOCK8 immunodeficiency is a consequence of an inability to collaborate with T cells, which is particularly limiting when access to antigen is reduced. These findings provide insights into how antigen abundance and interactions between B and T cells determine the priming, survival, and fate of antigen-activated B cell subsets that are essential for humoral immunity.

## Materials and Methods

### Study Design

This study was designed to characterize the role of DOCK8 in the maintenance of GC B cells using antigen-specific murine models. We used competitive adoptive transfer protocols and dual-nucleotide labeling approaches to interrogate the B cell intrinsic effects of DOCK8 on survival and proliferation of GC B cells. We performed flow cytometry analyses to characterize the effects of the loss of DOCK8 on the ability of GC B cells to present antigen to and collect help from T_FH_ cells. We used flow cytometry, scRNASeq analysis and immunohistochemical protocols to show that DOCK8-deficient GC B cells are arrested in the LZ at a stage where they fail to receive T cell help. We confirmed a B cell–intrinsic defect in receiving T cell help using ex vivo antigen-specific models that allowed us to finely titrate the amounts of antigen. Using the ex vivo model and low levels of antigen, we applied live-cell imaging techniques to demonstrate that DOCK8-deficient B cells make fewer T cell contacts and are less able to maintain B–T conjugates or regulate actin at the B–T synapse. The study design was not blinded and mice were randomly assigned to experiment groups without blinding of the investigators. However, the B–T conjugate microscopy data was also analyzed by researchers who were not involved in recording the imaging data and who were therefore blinded to the B cell genotypes. The *n* values reported for in vivo experiments in this study are biological replicates, unless otherwise indicated. The *n* values reported for ex vivo experiments in this study are either biological replicates or are data collected from cells isolated and pooled from two or more genetically identical donor mice, as indicated in legends. The number of mice per group, the number of independent replicates of the experiments and the statistical tests for analysis are included in the legends for each figure.

### Mice

All procedures involving mice were reviewed and approved by the University of Oxford ethical review committee and were performed in compliance with the Animals (Scientific Procedures) Act 1986, revised 2012 (ASPA). Mice of both sexes, 8–12 weeks of age, were used in the study and were age-matched within each experiment. *Dock8*^wt/wt^ and DOCK8-null *Dock8*^cpm/cpm^ mice were bred on a CD45.2^+^ or CD45.1^+^ C57BL/6-Tg(IghelMD4)4Ccg/J (MD4) background. C57BL/6-Tg(BCL2)22Wehi/J transgenic (BCL2.Tg) and C57BL/6-Tg(UBC-GFP)30Scha/J (GFP^+^) mice were crossed to *Dock8*^wt/wt^ or *Dock8*^cpm/cpm^ mice to generate mice with B cell–specific BCL2 expression or ubiquitous GFP expression or both. *Dock8*^wt/wt^ and *Dock8*^cpm/cpm^ MD4 mice were also crossed to B10.BR-H2k2 H2-T18a/SgSnJ to generate I-A^k^ or I-A^k^/I-A^b^–expressing mice for HEL peptide-MHC class II loading studies. B6.Cg-Tg(TcraTcrb)425Cbn/J (OTII) mice with or without GFP and WT CD45.1^+^ or CD45.2^+^ C57BL/6 mice were bred in-house.

### Antigens, antibodies, and activation reagents

Anti-DEC205, anti-DECOVA, anti-DECOVA-E333A (-EA) (negative control), ISOOVA, and anti-DECHEL antibodies cloned into pEE12.4 vector and OVAHEL antigen cloned into pEE14 vector were transiently expressed as His-tagged chimeric molecules in HEK293T cells and purified over Ni-NTA columns. SRBCs, SRBC-HEL and OVAHEL in RIBI adjuvant system at indicated doses were used as antigens for in vivo protocols. OVAHEL, HEL (L6876, Sigma) and HEL-EαGFP (gift from O. Bannard) were used as MD4 B cell antigens ex vivo at doses between 0.1–1000 ng/ml. LPS (L7770, Sigma), IL-4 (574304), IL-21 (574304), anti-CD28 (102116), anti-CD40 (102812) (BioLegend) and anti-IgM F(ab′)_2_ (715-006-020-JIR, Jackson Immuno Research) were used at indicated amounts.

### Adoptive transfers, immunization and antibody treatment

MD4 B cells from the spleen and OTII CD4 T cells from the spleen and mesenteric lymph node (MLN) were enriched to ~90% purity by magnetic sorting on LS columns using standard Miltenyi kits (130-090-862 and 130-104-454) according to manufacturer’s instructions. The purity of sorted B and T cells was then assessed by flow cytometry. Two hundred thousand enriched CD45.2^+^ *Dock8*^wt/wt^ and *Dock8*^cpm/cpm^ MD4 B cells, with or without BCL2.Tg, were adoptively transferred into CD45.1^+^ or CD45.2^+^ recipients, either alone or mixed at indicated ratios with CD45.1^+^ *Dock8*^wt/wt^ MD4 B cells, one day prior to immunization. Mice were immunized intraperitoneally (I.P.) with HEL conjugated to 1×10^9^ SRBCs (SRBC-HEL). When 50 μg of OVAHEL, mixed with RIBI Adjuvant System (Sigma, S6322) according to manufacturer’s protocol, was used for I.P. immunization, 1×10^5^ CD4 OTII T cells were also adoptively transferred. GC status was assessed on indicated days post-immunization. All antibodies were administered to mice I.P. at 10 μg per mouse 24 and 48 hours before analysis. Since activated and GC B cells, specifically LZ centrocytes, upregulate expression of the scavenger receptor DEC205 (9, 15, 28, 29), we used an OVA-conjugated anti-DEC205 antibody (anti-DECOVA) to assess BCR-independent delivery and presentation of antigen (30). To measure cognate antigen-dependent proliferation of B and T cells in vivo at 72 hours, sorted MD4 B cells were labeled with CellTrace Violet (CTV), incubated ex vivo with indicated amounts of antigen on ice for 25 min, and then washed three times with cold PBS to remove excess unbound antigen. Labeled B cells were then mixed with cognate CTV-labeled WT CD4 OTII T cells before they were transferred into recipients. Activation and proliferation were analyzed at 72 hours by flow cytometry.

### Proliferation and cell cycle measurements

In vivo GC B cell proliferation was assayed by measuring bromodeoxyuridine (BrdU) and/or 5-ethynyl-2′-deoxyuridine (EdU) incorporation. Mice were intravenously administered 2 mg of EdU (900584, Sigma) either 1.5 hours (short interval) or 4 hours (long interval) prior to the endpoint of the experiment. Both groups then received a further 3 mg of BrdU (B5002, Sigma) 30 min prior to the splenic GC assessment. Splenic single-cell suspensions were stained for surface markers before processing with BrdU flow kit (BD Biosciences), Click-iT EdU-Pacific Blue kit (Thermo Fisher), and SYTOX AADvanced Ready Flow Reagent according to manufacturers’ instructions. Cells were labeled with 5 μM CTV, CellTrace Yellow, CellTrace FarRed, or CellTrace CFSE (Thermo Fisher) and dilution of the CellTrace label was used to assess ex vivo proliferation of B and T cells at indicated time points. For Fig. 5D, the percentage of T cells within each CTV dilution peak was used to calculate the proportion of T cells that underwent at least one cell division, at least two cell divisions, etc.

### Flow cytometry

Single-cell suspensions were prepared from mouse MLN or spleens and stained as previously described (56). Briefly, tissue was mechanically disrupted using a sterile 3 ml syringe plunger. Dissociated cells were passed through a sterile 70 μm cell strainer and centrifuged at 300 x *g* for 10 minutes at 4°C. Cells were resuspended in RPMI and counted using a hemocytometer. PBS or HBSS with 20 mM HEPES, 10% FCS, and 0.05% sodium azide was used as a flow cytometry staining buffer. To stain cell surface markers, cells were incubated on ice for 25 min with appropriate dilutions of antibodies or antigens, as necessary. Antibody clones, sources, fluorescent labels and dilutions are listed in data S3. Near Infrared and Aqua dyes from BioLegend Zombie Live/Dead cell kits were used to identify dead cells. Cells were incubated with HEL (Sigma) at a concentration of 250 ng/ml followed by detection with HyHEL9 to identify HEL^+^ cells. For intracellular staining, BD CytoFix, CytoFix/CytoPerm, and Perm/Wash Buffer I were used according to the manufacturer’s instructions. Eα(52-68) peptide–bound I-A^b^ was assayed using the Y-Ae antibody (33) and HEL(46-61) peptide–bound I-A^k^ was measured using the C4H3 antibody (34). All flow cytometry data were recorded on a BD FACSCanto™ 10-Color flow cytometer using the BD FACSDiva software and analyzed using BD FlowJo.

### scRNASeq sample preparation

Magnetically enriched BCL2.Tg CD45.2^+^ *Dock8*^wt/wt^ or *Dock8*^cpm/cpm^ and CD45.1^+^ *Dock8*^wt/wt^ MD4 splenic B cells were adoptively transferred into CD45.1^+^ WT recipients before immunization with SRBC-HEL. On day 8, splenic B cells were first enriched by magnetic sorting on LS columns using standard Miltenyi kits. Purified B cells from three mice per group were multiplexed by hashtag-labeling with BioLegend TotalSeq-B antibodies B0301 #155831, B0302 #155833, B0303 #155835, B0304 #155837, B0305 #155839, B0306 #155841 before sorting CD45.1^+^ *Dock8*^wt/wt^ and CD45.2^+^ BCL2.Tg *Dock8*^wt/wt^ or *Dock8*^cpm/cpm^ MD4 HEL^+^ CD95^+^ GC B cells by flow cytometry using the BD FACSAria™ Fusion Cell Sorter. Multiplexed CD45.1^+^ and CD45.2^+^ GC B cell samples were processed using the 10X-Genomics scRNASeq sequencing platform. 5,430 CD45.1^+^ *Dock8*^wt/wt^ MD4 cells and 24,444 CD45.2^+^ *Dock8*^wt/wt^ and *Dock8*^cpm/cpm^ BCL2.Tg MD4 cells were captured and sequenced as two independent samples.

### scRNASeq data analysis

Single cell sequencing outputs were mapped to transcriptome mm10-2020-A and quantified with 10XGenomics CellRanger v5.0.1. The EmptyDroplets algorithm was used to call cells and a Seurat object was created from the gene expression (RNA) and hashtag oligo (HTO) libraries by filtering on cell barcodes identified in the RNA assay via CellRanger. Seurat v4.0 or higher R packages were used for further analyses (57). The HTO assay was CLR-normalized and demultiplexed using HTODemux to identify doublets, singlets and negative cells. Samples were filtered on the RNA assay for mitochondrial genes (<10%) and number of features (>100 and <5500) with 3,159 CD45.1^+^ and 12,758 CD45.2^+^ single cells available for downstream analyses.

The CD45.1^+^ and CD45.2^+^ datasets were integrated and the RNA assay in the integrated sample of 15,917 single cells was normalized using SCTransform before guided clustering, dimensionality reduction and data visualization. Differential RNA expression analysis and GSEA were performed on log-normalized or raw RNA counts using either FindMarkers within Seurat or muscat v1.4.0b (58), respectively. Publicly available Gene Expression Omnibus (GEO) datasets used for GSEA are indicated in figs. S1 and S2. Cells were assigned to specific GC states (LZ, DZ) or cell cycle states (G1/S/G2M) by scoring cells based on their similarity with gene expression profiles distinguishing these states using previously reported transcriptome data (59–61). Pseudotime scores and trajectory were analyzed using the Monocle package (62).

Six clusters identified at resolution 0.1 and 19 clusters identified at resolution 0.9 were assigned to specific GC cell subsets based on previously described gene expression profiles. Heatmap for top5 DEG and the top20 marker list (data S1) were used for preliminary identification of each cluster. At resolution 0.9, clusters were further annotated using landmark genes as follows: four PC/PB clusters characterized by higher expression of *Ighm, Igkc, Jchain, Prdm1*, and *Sdc1*, one T cell cluster, nine GC B cell clusters enriched for markers such as *Fas, Mef2b, Efnb1*, and *Bcl6* expression, and five early/activated/memory-like clusters. The GC B cell clusters included four DZ clusters, with markers of proliferation and somatic hypermutation, namely, *Pcna, Ezh2, Cxcr4*, and *Aicda*, and five LZ clusters, with higher expression of transcripts for a T cell–mediated selection/activation signature such as *Cd83, Cd86, Icam1, Slamf1*, and *Cd40*. DEG between the five LZ clusters were used to further characterize LZ2 and LZ3 as expressing activation markers: *Cd83, Cd86, Akt1, Pik3ap1, Pten*, and *Cd69*; LZ2 enriched for markers of recent T cell help: *Slamf1, Cd40, Icam1, Icosl, Batf, Nfkbid, Nfkbia*, and *Rel*; and LZ3 and LZ4 enriched for expression of DZ or cyclic re-entry markers: *Cxcr4, Foxo1, Aicda, Ccnd3*, and *Rhoh*. Memory precursor markers *Bach2, Bcl6, Klf2*, and *Zbtb20* were higher in LZ3, LZ4, and LZ-to-PrePC_Memory clusters. PC/PB markers *Ighm, Igkc, Jchain, Xbp1, Prdm1*, and *Sdc1* were high in LZ-to-PrePC_Memory.

### Ex vivo culture system

MD4 splenic B cells and CD4 T cells from spleens and MLN were purified by magnetic sorting on LS columns using standard Miltenyi kits according to the manufacturer’s protocol. Cells were cultured in RPMI-1640 medium with 100 mM non-essential amino acids, 20 mM HEPES buffer, 10% FCS, 100 U/ml of penicillin, 100 μg/ml of streptomycin, 2 mM L-glutamine, and 100 mM 2-mercaptoethanol. Unless otherwise stated, 2×10^5^ B cells and 1×10^5^ T cells were labeled with cell proliferation dyes and cultured for indicated time periods and with indicated antigens/reagents in U-bottom 96-well plates (Greiner) at 37°C and 5% CO_2_.

### Antigen-processing and conjugate-formation assays

For antigen-processing assays, splenic B cells were loaded with CellTrace dye, incubated with antigen for 4 hours and washed before staining for surface pMHC complexes. For the flow-based conjugate formation assay, *Dock8*^wt/wt^ and *Dock8*^cpm/cpm^ MD4 B cells were labeled with CellTrace dye and cultured with indicated antigens and WT OTII CD4 T cells for 16 hours. Conjugates were fixed with CytoFix (BD) on ice for 15 min before washing and staining for surface markers, prior to analysis by flow cytometry.

### Microscopy and live-cell imaging

For live-cell imaging assays of B–T conjugate formation, *Dock8*^wt/wt^ and *Dock8*^cpm/cpm^ MD4 splenic B cells were sorted and labeled with indicated CellTrace dyes. B cells were incubated with OVAHEL antigen for 6–12 hours before washing and transferring 2×10^5^ cells each to fibronectin-coated chamber slides. CellTrace dye-labeled or GFP^+^ WT OTII CD4 T cells and warm RPMI-1640 medium without phenol red and with 20 mM HEPES were added to each well prior to imaging. Conjugate formation was monitored in real-time using a Leica SP8 WLL SMD-X point scanning confocal microscope. Images were recorded at 37°C every 30 s for 2 hours at a time, for a total period of 8–10 hours and across 9–18 frames. Images were acquired using the Leica Application Suite X (LASX) software, a 60X 1.4NA oil immersion objective, and 405-nm, 488-nm, 561-nm, and 470–670-nm lasers. Inbuilt PMT and HyD detectors and the spectral detector array were used to record data. All microscopy imaging data were analyzed using the Fiji (ImageJ) software package (63).

To assess actin cytoskeleton status in B–T cell conjugates, MD4 splenic B cells were differentiated to GC-like CD95^+^GL7^+^ B cells with 5 μg/ml of anti-CD40, 20 ng/ml of IL-4, and 20 ng/ml of IL-21 for 48–72 hours at 37°C and 5% CO_2_. GC-like B cells were incubated with 10 ng/ml of OVAHEL antigen for 6–12 hours, loaded with SiR-actin and CellTrace dyes according to manufacturer’s instructions and mixed with WT OTII CD4 T cells. B–T cell conjugates on fibronectin-coated surfaces were imaged at 37°C and 5% CO_2_ in RPMI-1640 medium without phenol red and buffered with 20 mM HEPES as described above. For each B–T cell conjugate, data were recorded either with *x*-, *y*-, and *z*-coordinates or as a time-series with *x*- and *y*-coordinates. Leica SP8 FALCON point-scanning confocal microscope equipped with a LASX module, a 60X 1.3NA water immersion objective, four HyD detectors, and 405-nm, 488-nm, 561-nm, and 633-nm lasers were used to record data B–T cell conjugates. Data were analyzed using the Fiji (ImageJ) software with the 3D Viewer plugin (63). The number of actin projections at the B–T cell interface from 13 *Dock8*^wt/wt^ and 20 *Dock8*^cpm/cpm^ MD4 B cells were independently enumerated by a researcher who was not involved with the recording of the data and was therefore blinded regarding the B cell genotypes.

For the B cell–spreading assay, *Dock8*^wt/wt^ and *Dock8*^cpm/cpm^ MD4 splenic B cells were activated with 5 μg/ml of anti-IgM, 5 μg/ml of anti-CD40, 20 ng/ml of IL-4, and 20 ng/ml of IL-21 for 48–72 hours to induce a GC-like phenotype. CD95^+^ GL7^+^ MD4 B cells were allowed to adhere to and spread on anti-CD44-coated chamber slides before fixing cells with BD Cytofix/Cytoperm. Cells were washed and stained with Flash Phalloidin Red 594 and an anti-tubulin-beta 3 antibody. Images were recorded on a Leica SP8 FALCON point-scanning confocal microscope using a 60X 1.3NA water immersion objective, four HyD detectors, and 405-nm, 488-nm, 561-nm, and 633-nm lasers. Data were recorded using the LASX software and analyzed using the Fiji (ImageJ) software (63).

### Tissue immunofluorescence

CD45.1^+^ *Dock8*^wt/wt^ recipients were immunized with SRBC-HEL 1 day after transfer of *Dock8*^wt/wt^ or *Dock8*^cpm/cpm^ BCL2.Tg GFP^+^ MD4 splenic B cells. Spleens were collected on day 8, fixed, dehydrated, and frozen before sectioning at 10–20 μm. Sections were processed as previously described (64) and stained for expression of IgD and the follicular dendritic cell marker FDC-M2. Prolong Glass or Prolong Diamond Antifade mountant were used to mount samples prior to storing or imaging. Images were recorded on a Leica SP8 FALCON point-scanning confocal microscope using a 20X 0.7NA air objective and a 60X 1.3NA water immersion objective with four HyD detectors, and 405-nm, 488-nm, 561-nm, and 633-nm lasers. Data were recorded using the LASX software and analyzed using the Fiji (ImageJ) software (63).

### Statistical and other data analysis

Flow cytometry data were recorded using BD FACSDiva and analyzed with BD FlowJo. Microscopy imaging data were recorded using the Leica proprietary LASX software and analyzed using open-source Fiji (ImageJ) software (63). Microsoft Excel and GraphPad Prism 8 or higher were used for statistical analyses. All data values are represented in the figures where possible and are provided in the supplementary materials (data S4). The figure legends and the supplementary materials provide information on sample size, number of independent replicates, and confidence intervals. Unpaired *t* tests with Welch’s correction were used to compare two groups and ordinary one-way or two-way analysis of variance (ANOVA) was used when comparing three or more groups for statistical testing. Post-hoc tests were performed when an overall statistically significant *P*-value was reported for the ANOVA. *P*-value thresholds are indicated in figure legends. A result was considered significant if the *P*-value was less than 0.05. Testing parameters were corrected for multiple *t* tests using the Holm–Šídák method and for ANOVA using Tukey’s or Šídák post-hoc multiple-comparisons tests.

## Supplementary Material

data files S1 to S4

movies S1 to S11

supplementary materials (including fig. S1 to s9)

## Data Availability

Single-cell RNA-sequencing data generated during this study (Fig. 2, and figs. S1 and S2) are available at the Gene Expression Omnibus database under accession number GSE269130. Data from microscopy experiments (Fig. 7 and figs. S3 and S8) are available in the main or supplementary figures or in the supplementary materials (movies S1 to S11). Experimental protocols and information about reagents used in this study are available in the Materials and Methods section, in data S3, or in indicated references. No new custom algorithms were generated in this study. Publicly available R packages were used for scRNASeq data analyses, including Seurat version 4.0 or higher (57), muscat v1.4.0b (58) and Monocle (62), and are indicated in the Materials and Methods section. All other data required to evaluate the conclusions of this study are available in the paper or in the supplementary materials, including raw data in data S4.

## One-Sentence Summary

B cells activated by low amounts of antigen require DOCK8 to acquire survival and differentiation signals via T cell help.
