# Supplementary material for: B cells require DOCK8 to elicit and integrate T cell help when antigen is limiting
Source: Sci Immunol. Author manuscript; Available in PMC 2024 Aug 22. (PMC7616390; doi:10.1126/sciimmunol.add4874)
Supplement: supplementary materials (including fig. S1 to s9) [file EMS198076-supplement-supplementary_materials__including_fig__S1_to_s9_.pdf]

Supplementary Materials for

B cells require DOCK8 to elicit and integrate T cell help when antigen is limiting

Mukta Deobagkar-Lele, Greg Crawford, Tanya L. Crockford, Jennifer Back, Rose Hodgson, Aneesha Bhandari, Katherine R. Bull, Richard John Cornall

Corresponding authors: mukta.deobagkar@ndm.ox.ac.uk (M.D.-L.); richard.cornall@ndm.ox.ac.uk (R.J.C.)

For the purpose of open access, the authors have applied a Creative Commons attribution (CC BY) public copyright license to any Author Accepted Manuscript (AAM) version arising from this submission.

Accepted for publication in ***SCIENCE IMMUNOLOGY***

DOI: 10.1126/sciimmunol.add4874

The PDF file includes:

Figs. S1 to S9
Captions for Movies S1 to S11
Captions for Data S1 to S4

Other Supplementary Material for this manuscript includes the following:

Movies S1 to S11
Data S1 to S4
MDAR Reproducibility Checklist

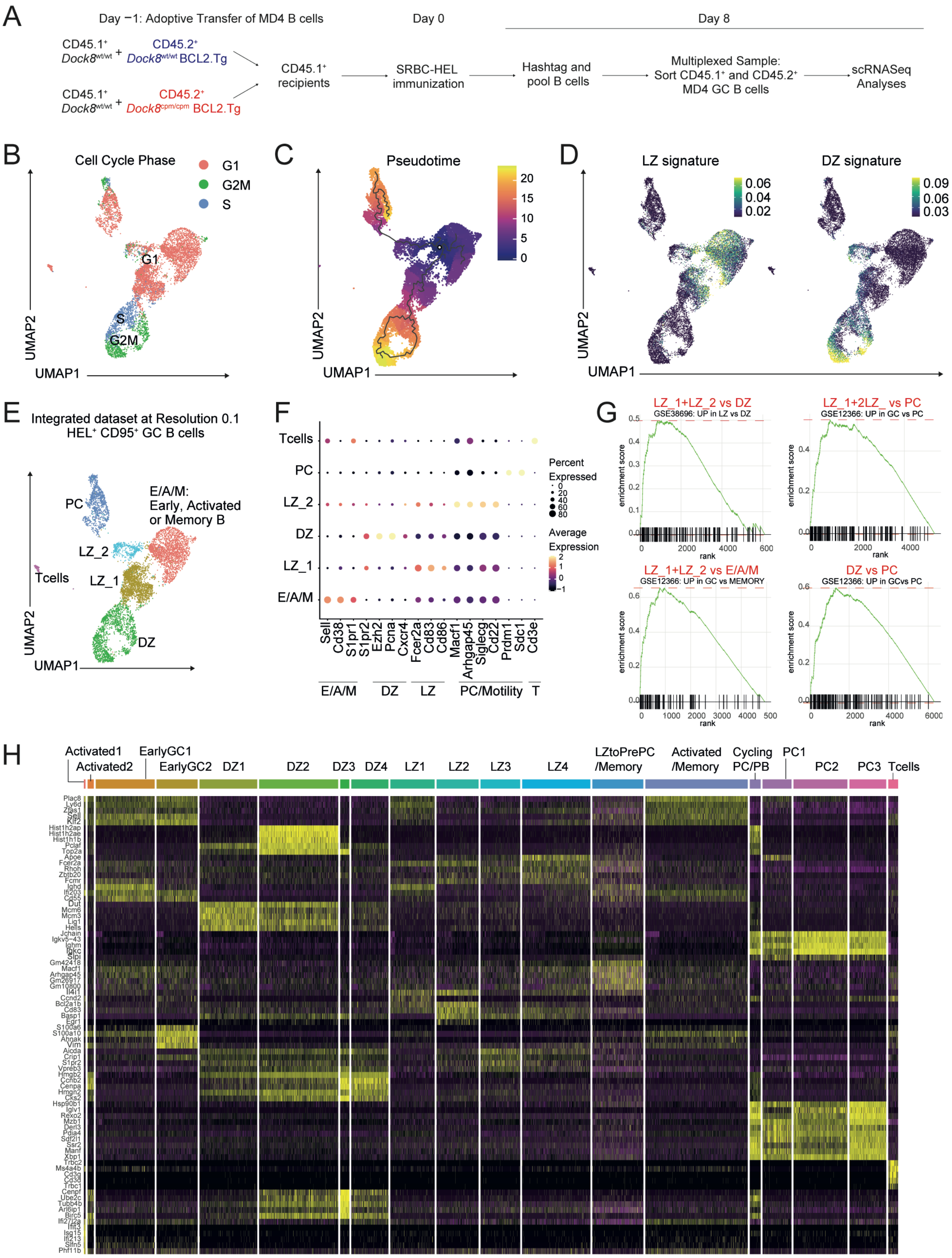

Fig. S1. Unsupervised clustering of integrated scRNASeq data to identify GC subsets (related to Fig. 2). (**A**) Schematic for scRNASeq analysis of CD45.1^{+} (*Dock8*$^{\text{wt/wt}}$) and CD45.2^{+} BCL2.Tg (*Dock8*$^{\text{wt/wt}}$ or *Dock8*$^{\text{cpm/cpm}}$) splenic HEL^{+}IgM^{a+}CD95^{+} MD4 GC B cells. Three mice each were immunized with SRBC-HEL 1 day after adoptive transfer of CD45.1^{+} (*Dock8*$^{\text{wt/wt}}$) and CD45.2^{+} BCL2.Tg (*Dock8*$^{\text{wt/wt}}$ or *Dock8*$^{\text{cpm/cpm}}$) MD4 B cells. GC B cells were sorted by flow cytometry, hashed and pooled for single-cell sequencing on day 8. (**B**) UMAP

of the integrated scRNASeq dataset shows the relative contribution of cell-cycle scores on a per-cell basis. (**C**) Monocle-derived Pseudotime scores for the integrated GC B cell dataset projected onto a UMAP. (**D**) Enrichment score for gene signatures distinguishing LZ and DZ states. (**E**) UMAP of the integrated dataset with six clusters at 0.1 resolution. (B to E) Each dot on the UMAPs indicates a single cell and colors indicate either cluster identity or scores. (**F**) Selected landmark DEGs to inform cluster identity. (**G**) GSEA between the six clusters to confirm their identities using previously published gene signatures, as indicated. (**H**) Heatmap of the top5 DEG for 19 clusters at 0.9 resolution (see Fig. 2, A and D, for cluster label colors).

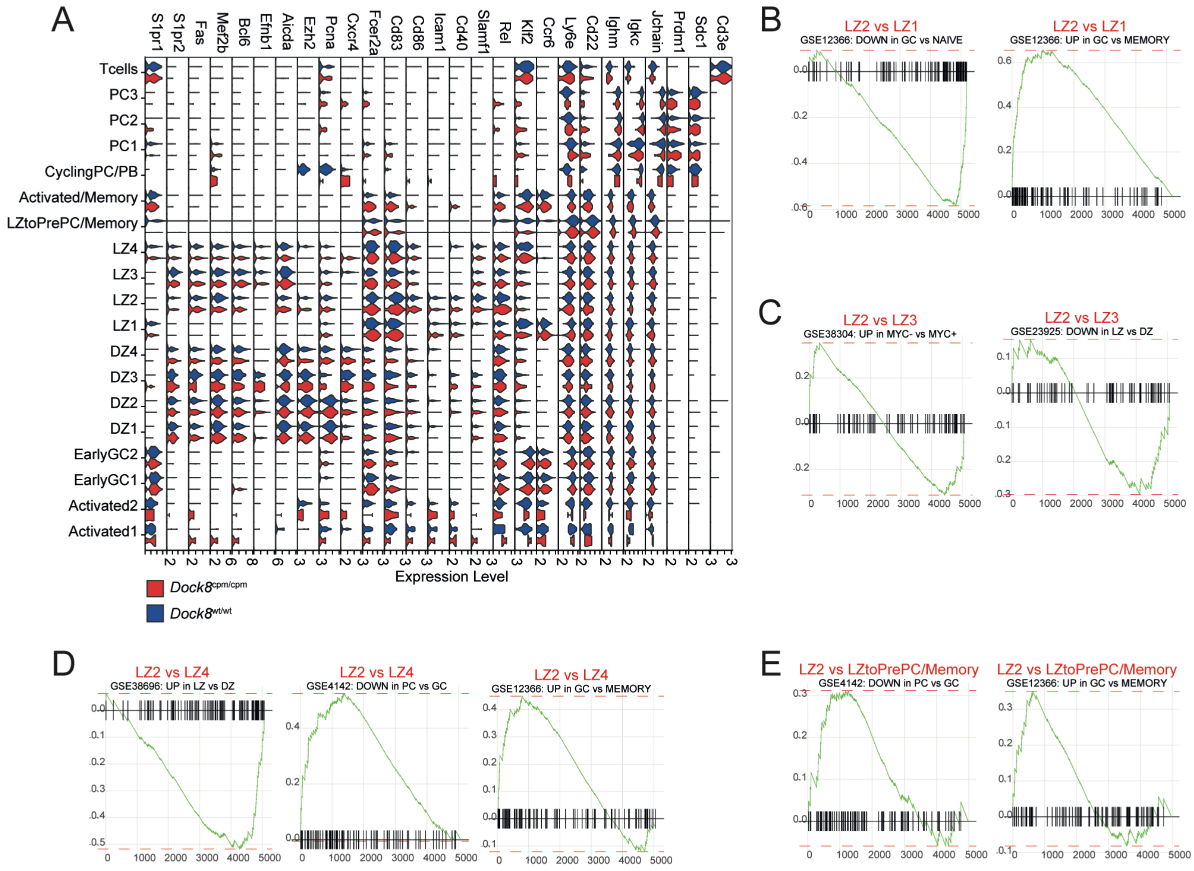

Fig. S2. Deep immunophenotyping of GC B cells using scRNASeq data (related to Fig. 2). (**A**) Stacked violin plots of differential expression between the *Dock8*$^{wt/wt}$ and *Dock8*$^{cpm/cpm}$ groups of the landmark genes across the 19 clusters at 0.9 resolution (see Fig. 2B). (**B** to **E**) GSEA between LZ2 and the other four LZ clusters to confirm their relative LZ, DZ, plasma cell or memory B cell signatures using previously published datasets, as indicated: (B) LZ2 versus LZ1 (the two clusters with highest *Cd83* expression); (C) LZ2 versus LZ3; (D) LZ2 versus LZ4; and (E) LZ2 versus LZtoPrePC/Memory.

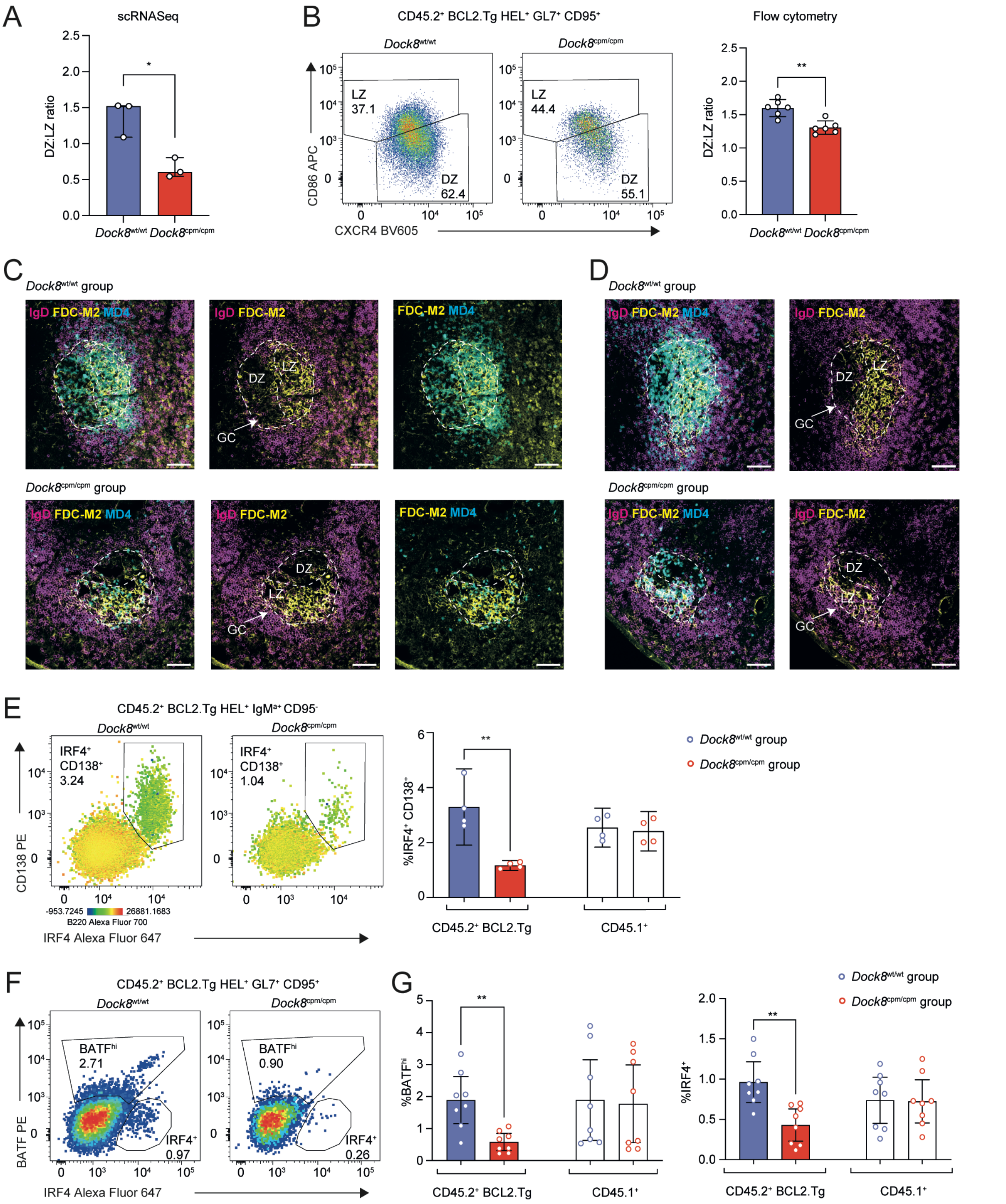

Fig. S3. DOCK8-deficient GC B cells are blocked in the LZ and do not receive T cell help or differentiation signals. (**A**) Quantification of DZ:LZ ratio of CD45.2^{+} BCL2.Tg *Dock8*$^{wt/wt}$ and *Dock8*$^{cpm/cpm}$ splenic GC B cells as calculated from the scRNASeq dataset (see: Fig 2, D and E, and fig. S1, D and E). (**B**) Representative flow cytometry plots for DZ and LZ status (left), and quantification of the DZ:LZ ratios of CD45.2^{+} BCL2.Tg *Dock8*$^{wt/wt}$ and *Dock8*$^{cpm/cpm}$ splenic GC B cells (right). Data are representative of three experiments with 4–6 mice per group. (**C** and **D**) *Dock8*$^{wt/wt}$ or *Dock8*$^{cpm/cpm}$ CD45.2^{+} BCL2.Tg GFP^{+} MD4 spleen B cells were adoptively transferred into CD45.1 recipients before immunization with SRBC-HEL. Representative images for the distribution of GFP^{+} MD4 B cells (cyan), FDC-M2 expression in the LZ (yellow) and IgD expression on non-GC B cells (magenta) in the spleens of mice

from *Dock8*$^{wt/wt}$ (top panels) or *Dock8*$^{cpm/cpm}$ (bottom panels) groups on day 8. Dashed lines indicate the GC, LZ, and DZ regions, and scale bars represent 50 µm. (**E** to **G**) CD45.2^{+} BCL2.Tg *Dock8*$^{wt/wt}$ or *Dock8*$^{cpm/cpm}$ GC spleen B cells on day 8 after adoptive transfer and immunization with SRBC-HEL. (E) Representative flow cytometry plots and quantified data for CD138^{+}IRF4^{+}B220^{low} PCs. Data are representative of two experiments with $n = 4$ mice per group. (F) Representative flow cytometry plots and (G) quantified data for BATF^{hi} and IRF4^{+} HEL^{+}GL7^{+}CD95^{+} splenic GC B cells on day 8. Data are pooled from two experiments with four mice per group. (A, B, E, and G) Symbols represent individual mice and bars indicate means and error bars denote 95% confidence intervals. Unpaired two-tailed *t* tests with Welch's correction were used for analysis with $^{*}P<0.05$ and $^{**}P<0.01$.

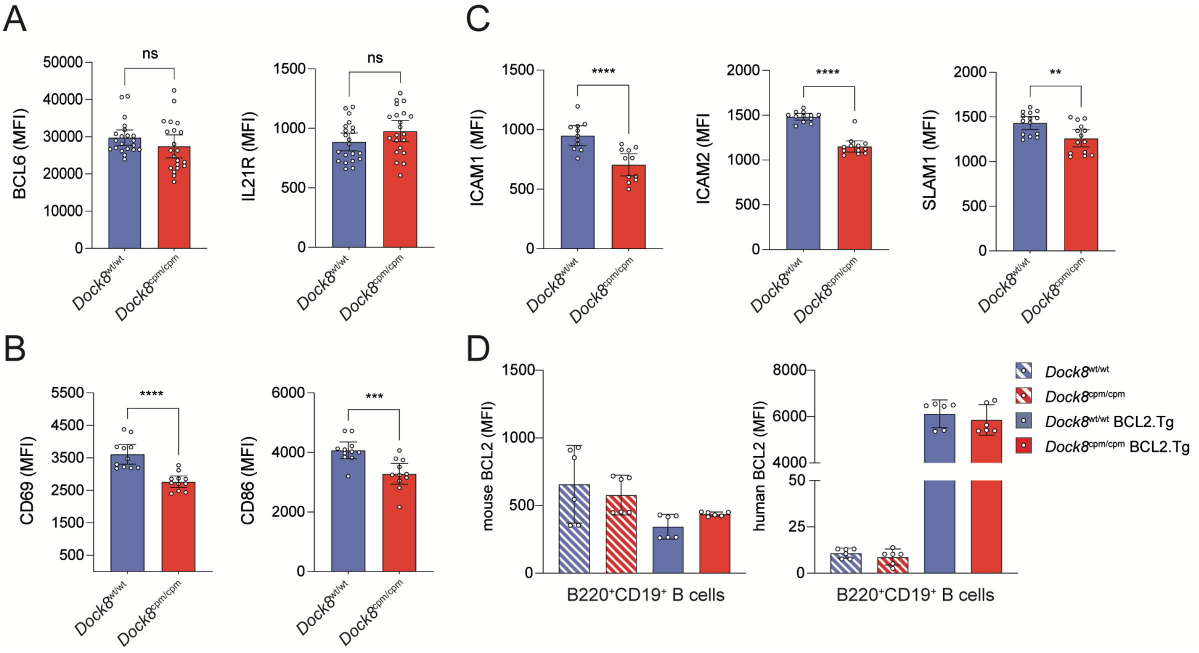

Fig. S4. Effect of DOCK8 on B cell expression of phenotypic, activation and survival markers. *Dock8*$^{wt/wt}$ or *Dock8*$^{cpm/cpm}$ BCL2.Tg GFP$^+$ MD4 spleen B cells were adoptively transferred into *Dock8*$^{wt/wt}$ C57BL/6 recipients before immunization with SRBC-HEL. (**A**) Surface expression of GC markers, (**B**) activation markers, (**C**) integrins and adhesion markers on CD95$^+$GL7$^+$IgM^{a+}GFP$^+$HEL$^+$ splenic GC B cells after 8 days. (A to C) Data are pooled from two to three experiments with n = 4–9 mice per group per experiment. Bars indicate means with 95% confidence intervals and symbols represent individual mice. Unpaired two-tailed t tests with Welch's correction were used for analysis with **P<0.01, ***P<0.001, and ****P<0.0001. (**D**) Levels of the mouse BCL2 and the transgenic human BCL2 protein in splenic B cells from *Dock8*$^{wt/wt}$ or *Dock8*$^{cpm/cpm}$ non-transgenic and BCL2.Tg mice. Data are pooled from two experiments with n = 3 mice per group per experiment. Bars show means, error bars indicate 95% confidence intervals and circles indicate data from individual mice. Ordinary one-way ANOVA with Tukey's multiple-comparison test was used for statistical analyses.

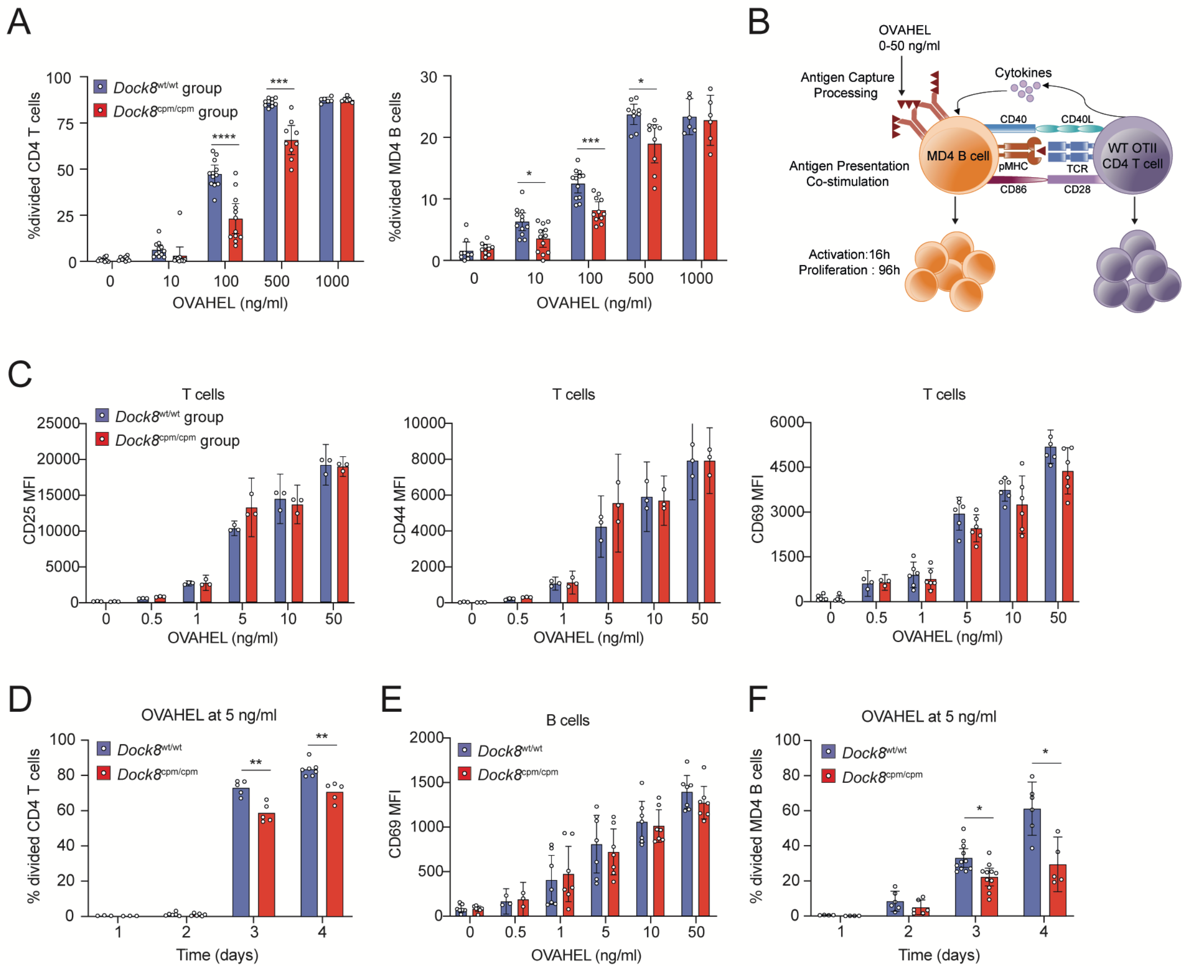

Fig. S5. DOCK8 is required for B and T cell proliferation when antigen is limiting. (**A**) CTV-labeled CD45.2^{+} *Dock8*$^{\mathrm{wt/wt}}$ or *Dock8*$^{\mathrm{cpm/cpm}}$ MD4 spleen B cells were loaded with indicated amounts of OVAHEL, mixed with CTV-labeled CD45.2^{+} OTII CD4 T cells and adoptively transferred into CD45.1^{+} recipients. Spleens were analyzed at 72 hours to assess proliferation of transferred CD45.2^{+} OTII CD4 T cells (left) and CD45.2^{+} MD4 B cells (right). Data are pooled from two experiments with n = 3–6 mice per group. (**B**) Illustration of the ex vivo model used to dissect B and T cell interactions. (**C**) Dose-dependent upregulation of activation markers on OTII CD4 T cells after 16 hours of culture with *Dock8*$^{\mathrm{wt/wt}}$ or *Dock8*$^{\mathrm{cpm/cpm}}$ splenic MD4 B cells and OVAHEL. (**D**) Proliferation of OTII CD4 T cells cultured with *Dock8*$^{\mathrm{wt/wt}}$ or *Dock8*$^{\mathrm{cpm/cpm}}$ MD4 B cells and 5 ng/ml of OVAHEL for indicated time points. (**E**) CD69 expression on OVAHEL-activated *Dock8*$^{\mathrm{wt/wt}}$ or *Dock8*$^{\mathrm{cpm/cpm}}$ MD4 B cells after 16 hours with OTII CD4 T cells. (**F**) Proliferation of *Dock8*$^{\mathrm{wt/wt}}$ or *Dock8*$^{\mathrm{cpm/cpm}}$ MD4 B cells at indicated time points after culture with OTII CD4 T cells and 5 ng/ml of OVAHEL. Data are pooled from N = 3–6 independent experiments with cells pooled from n = 2–6 mice per group. Symbols represent data from individual samples or mice, and bars show means with 95% confidence intervals. Data were analyzed by unpaired two-tailed t tests with Welch's correction and the Holm–Šídák method for multiple-comparisons, with *P<0.05, **P<0.01, ***P<0.001, and ****P<0.0001.

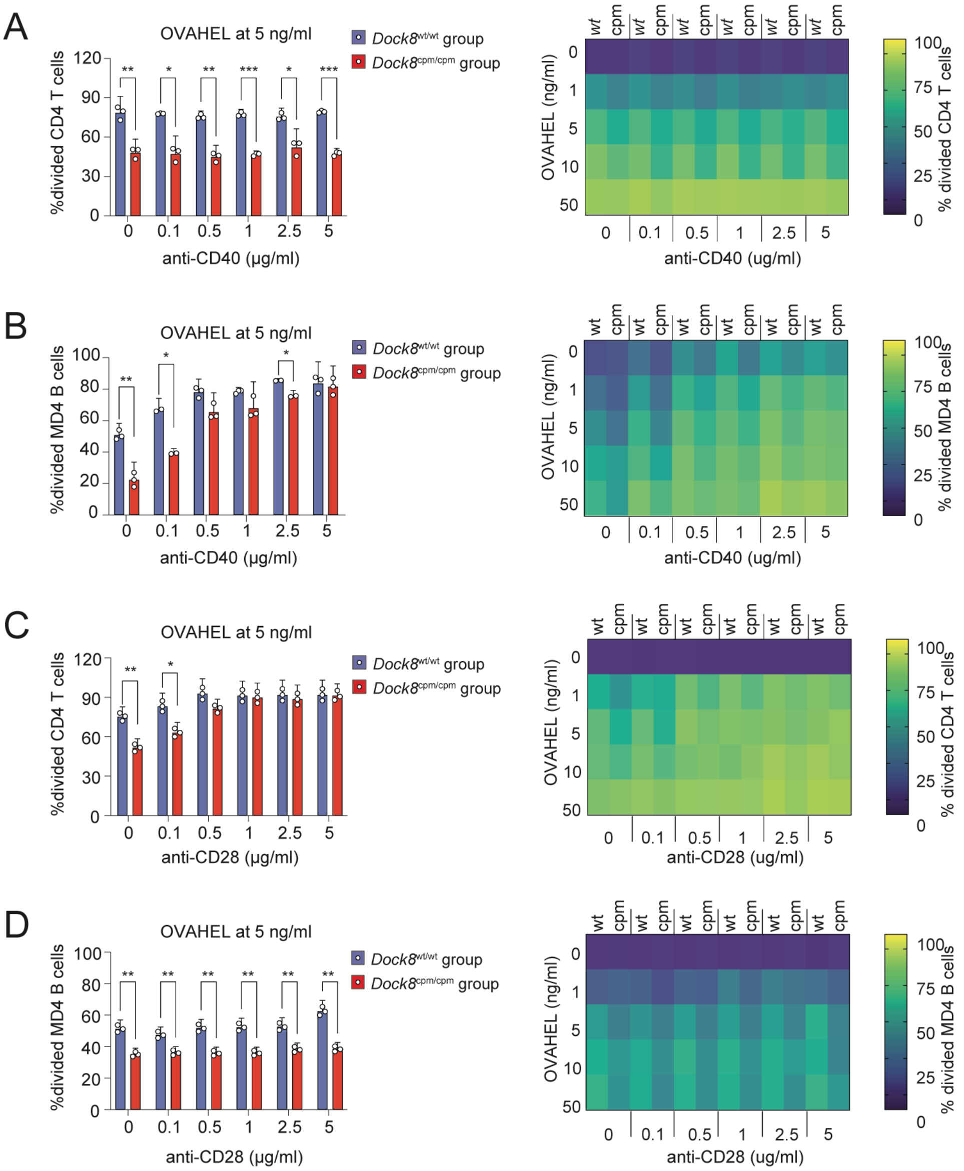

Fig. S6. Defective B–T cell costimulatory interactions at low antigen levels in the absence of DOCK8. (**A** to **D**) *Dock8*$^{wt/wt}$ or *Dock8*$^{cpm/cpm}$ splenic MD4 B cells and OTII CD4 T cells were cultured with indicated amounts of OVAHEL and anti-CD40 or anti-CD28 antibody for 96 hours. (A) T cell and (B) B cell proliferation at 96 hours with 5 ng/ml of OVAHEL and indicated amounts of anti-CD40 antibody (left) or heatmaps for (A) T cell and (B) B cell proliferation with indicated combinations of OVAHEL and anti-CD40 antibody (right). (C) T cell and (D) B cell proliferation at 96 hours with 5 ng/ml of OVAHEL and indicated amounts of anti-CD28 antibody (left) or heatmaps with indicated combinations of OVAHEL and anti-CD28 antibody (right). Circles indicate individual samples, bars show means with 95% confidence intervals. Cells were pooled from two mice per group in each experiment. Data are pooled from $N = 2$–3 experiments (bar graphs) or 1–3 experiments (heatmaps). Unpaired *t* tests with Welch's correction and the Holm–Šídák method for multiple-comparisons were used for analyses with *$P<0.05$, **$P<0.01$, and ***$P<0.001$.

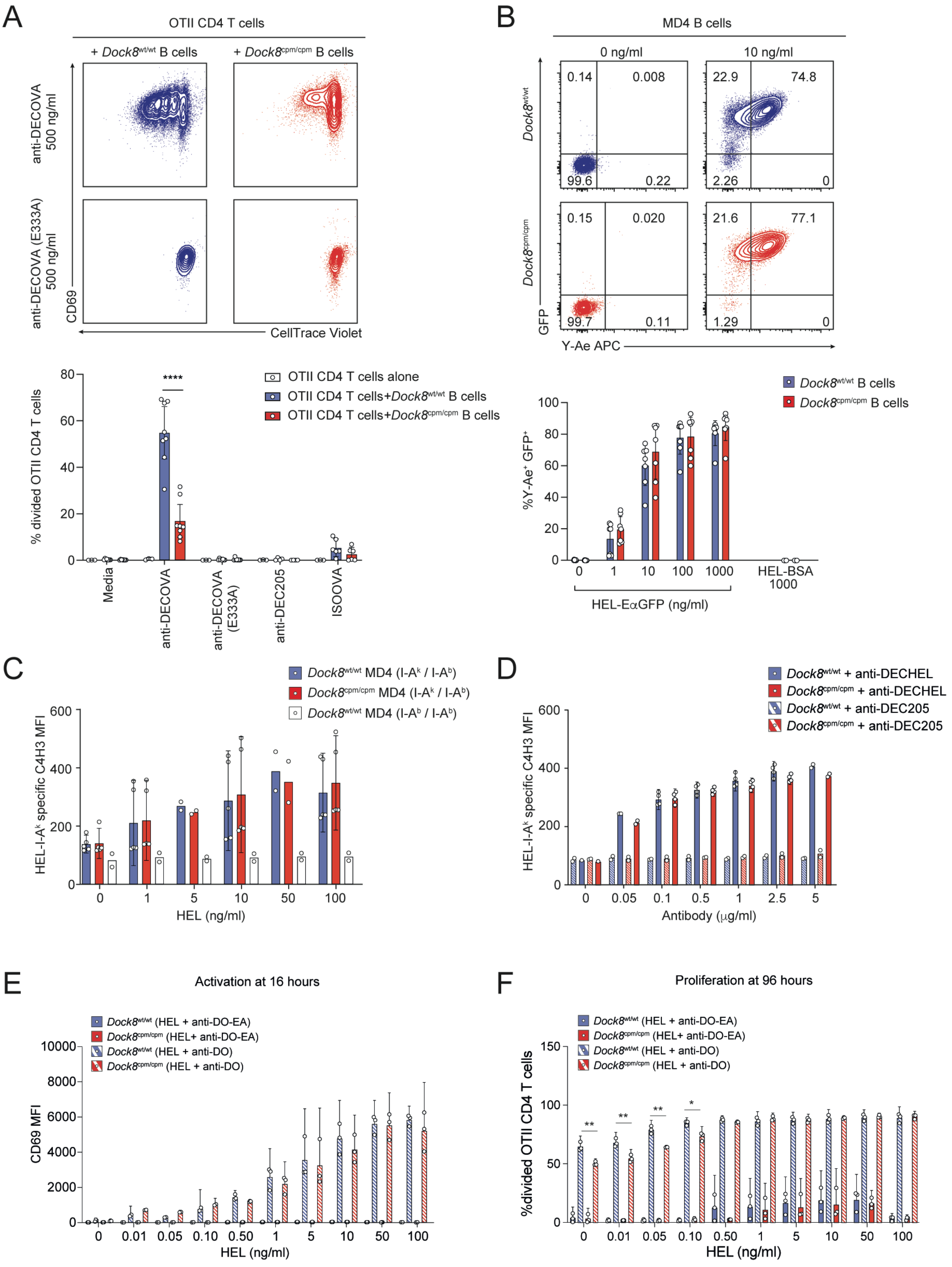

Fig. S7. B cells require DOCK8 to elicit T cell help but not for presentation of limiting amounts of soluble antigen. (**A**) Representative flow cytometry plots and pooled data from four experiments with two mice each for proliferation of OTII CD4 T cells cultured with *Dock8*$^{wt/wt}$ or *Dock8*$^{cpm/cpm}$ MD4 B cells and 500 ng/ml of indicated antibodies for 96 hours. Data analyzed using two-way ANOVA and Tukey's multiple-comparisons test with *****P*<0.0001. (**B**) Representative flow cytometry plots and pooled data for Eα(52-68) peptide bound to I-A^{b} on MD4 B cells after culture with HEL-EαGFP for 4 hours. Data are pooled from *N* = 3–7 independent experiments. (A and B) Circles denote data from individual mice and bars indicate means with 95% confidence intervals. (**C** and **D**) HEL(46-61) peptide bound

to I-A^k on the surface of (C) *Dock8*$^{wt/wt}$ I-A^b/I-A^b and *Dock8*$^{wt/wt}$ or *Dock8*$^{cpm/cpm}$ I-A^b/I-A^k MD4 B cells cultured with indicated doses of HEL for 4 hours, and (D) LPS-activated *Dock8*$^{wt/wt}$ or *Dock8*$^{cpm/cpm}$ B cells cultured with indicated amounts of anti-DECOVA or anti-DECHEL for 24 hours. Data are pooled from N = 2–5 experiments with circles indicating data pooled from two mice per sample. Bars indicate means with 95% confidence intervals. (**E** and **F**) Mixtures of OTII CD4 T cells with *Dock8*$^{wt/wt}$ or *Dock8*$^{cpm/cpm}$ MD4 spleen B cells, labeled with CTV and incubated with indicated HEL amounts and 10 μg/ml each anti-DECOVA (DO) or anti-DECOVA-E333A (DO-EA). Pooled data for (E) expression of CD69 on OTII CD4 T cells at 16 hours and (F) proliferation at 96 hours. Data are pooled from N = 2–3 experiments with cells pooled from n = 2–3 mice per sample. Bars show means with 95% confidence intervals and symbols indicate individual samples. Data analyzed using two-way ANOVA with Šídák's multiple-comparisons test, and *P<0.05 and **P<0.01.

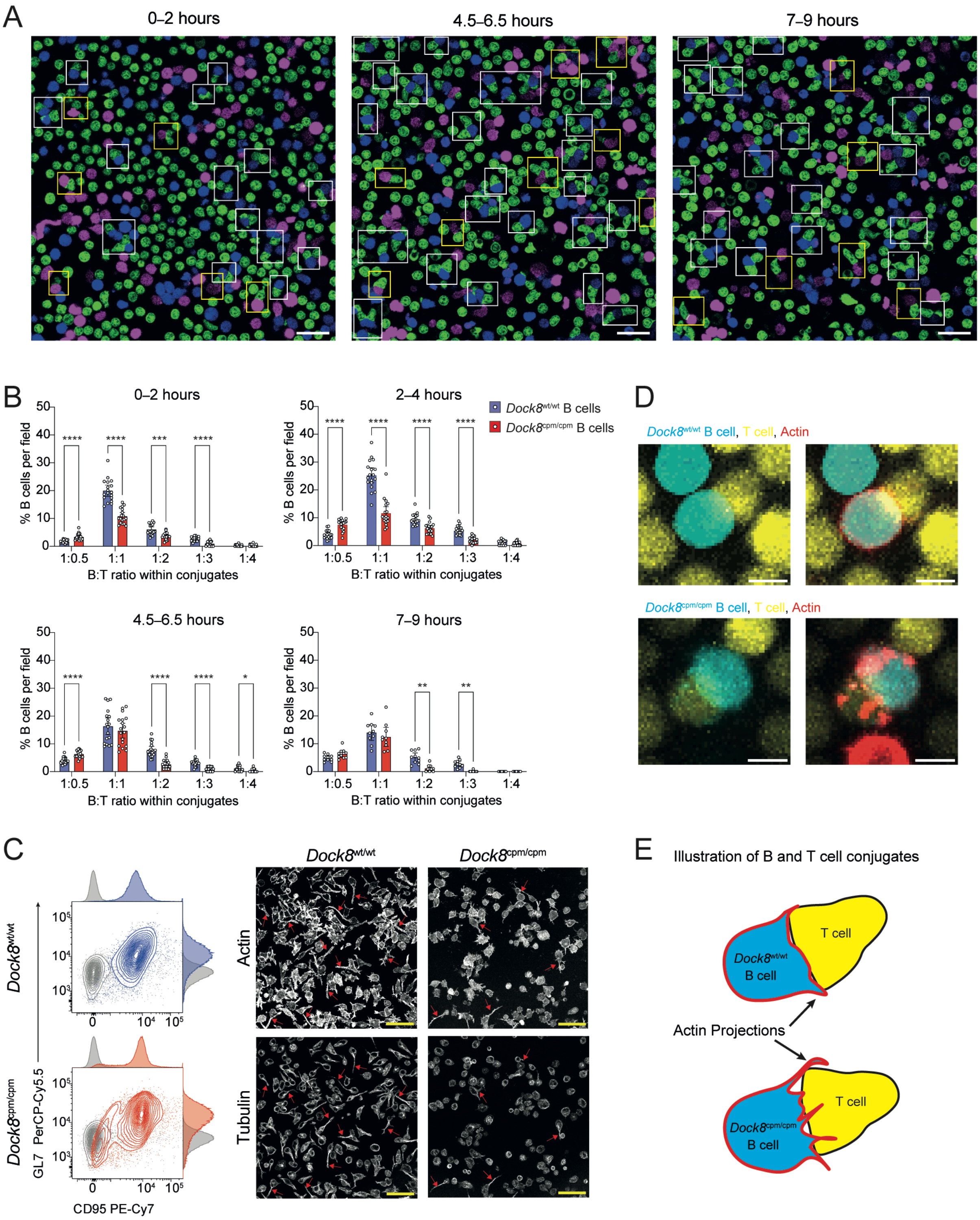

Fig. S8. DOCK8 modulates B–T cell conjugate formation and actin cytoskeleton when antigen is limiting. (**A**) Representative images for B–T cell conjugate formation in the presence of 10 ng/ml of OVAHEL at indicated time points. *Dock8*$^{wt/wt}$ MD4 B cells in blue with white boxes indicating conjugates, *Dock8*$^{cpm/cpm}$ MD4 B cells in magenta with yellow boxes indicating conjugates and OTII CD4 T cells in green. (**B**) Distribution of T cell conjugates with *Dock8*$^{wt/wt}$ or *Dock8*$^{cpm/cpm}$ MD4 B cells at indicated B:T ratios. Data are representative of three experiments with cells pooled from n = 2–3 mice per group. Symbols indicate the proportion of B cells in each field of view that form conjugates. Bars show means with 95% confidence intervals. Data were analyzed by unpaired Welch's t test and the Holm-

Šídák method for multiple comparisons, with *P<0.05, **P<0.01, ***P<0.001, and ****P<0.0001. (**C**) *Dock8*$^{wt/wt}$ or *Dock8*$^{cpm/cpm}$ splenic B cells, activated with anti-IgM, anti-CD40, IL-4, and IL-21 to induce CD95^{+}GL7^{+} GC-like phenotype, were allowed to adhere to anti-CD44 coated surfaces for 8–12 hours. Representative flow cytometry plots for expression of GC markers on naïve and activated B cells (left). Cells were fixed and stained for expression of actin and tubulin (right). Red arrows indicate B cell spreading on anti-CD44 coated surfaces. (**D**) Ex vivo generated GC-like *Dock8*$^{wt/wt}$ or *Dock8*$^{cpm/cpm}$ B cells (cyan) were incubated with 10 ng/ml OVAHEL for 16 hours, labeled with SiR-actin (red) and mixed with CTY-labeled OTII CD4 T cells (yellow). B–T conjugates were imaged as previously described. Representative images of B and T cell conjugates extracted from time course data. Scale bar indicates 20 µm for (A), 50 µm for (C) and 5 µm for (D). (**E**) Illustration of actin-rich extensions when *Dock8*$^{wt/wt}$ or *Dock8*$^{cpm/cpm}$ B cells form contacts with cognate CD4 T cells.

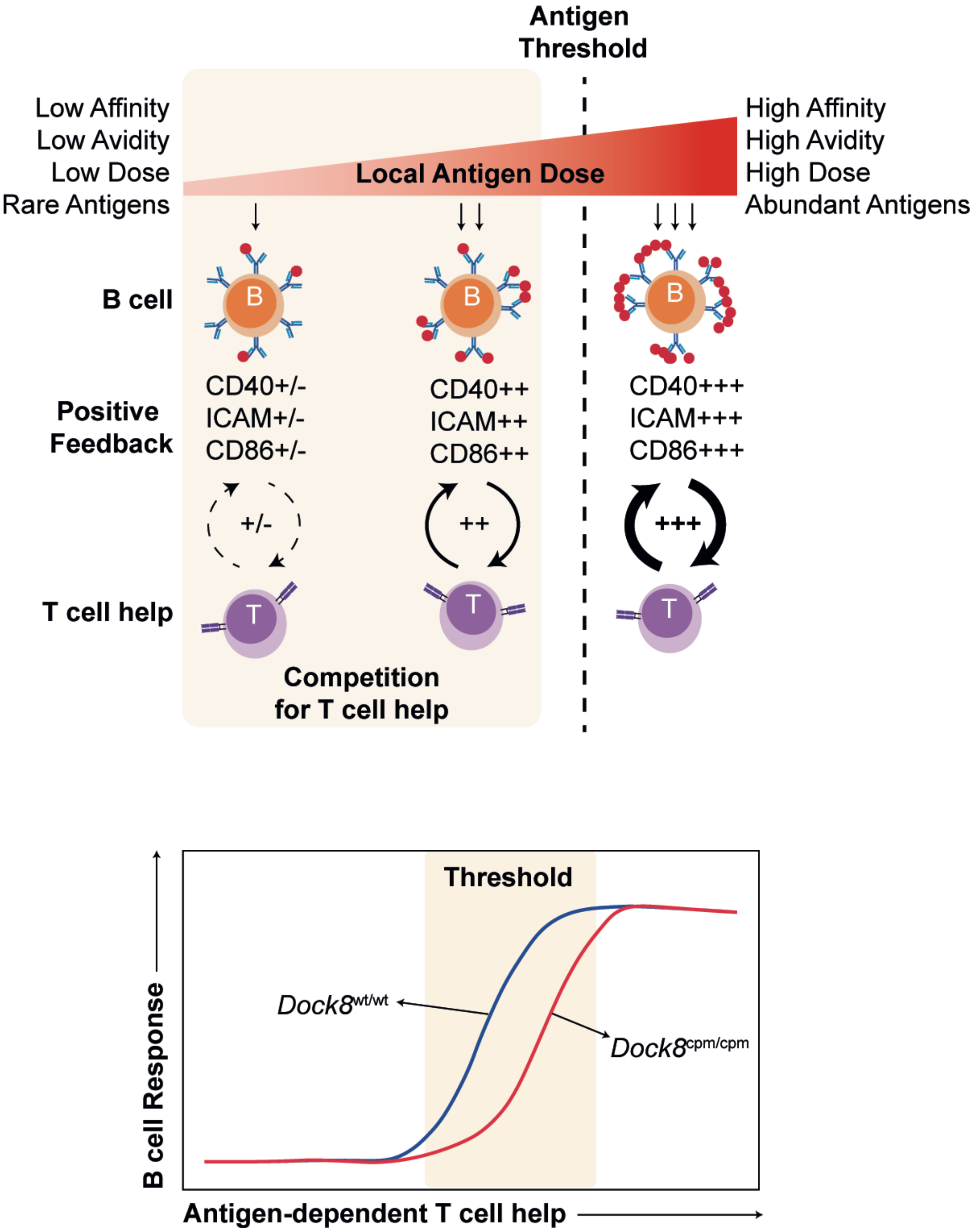

Fig. S9. Competition for T cell help within the GC is dependent on local antigen dose and modulated by DOCK8. Under physiological conditions, most antigens are of low affinity and abundance. Lower localized antigen levels within the GC elicit corresponding lower activation of B cells, reinforcing dependence on T cell help and ancillary pathways, such as those modulated by DOCK8. Maintaining antigen thresholds within the GC creates the competitive GC environment that supports selection and survival of the fittest B cell clones.

Movie S1. B–T_Conjugates_0–2hr.avi. *Dock8*$^{wt/wt}$ MD4 B cells (blue) and *Dock8*$^{cpm/cpm}$ MD4 B cells (magenta), activated overnight with 10 ng/ml of OVAHEL, forming conjugates with OTII CD4 T cells (green). Data recorded at 30-s intervals, over a period of 0 to 2 hours from the addition of T cells.

Movie S2. B–T_Conjugates_2–4hr.avi. *Dock8*$^{wt/wt}$ MD4 B cells (blue) and *Dock8*$^{cpm/cpm}$ MD4 B cells (magenta), activated overnight with 10 ng/ml of OVAHEL, forming conjugates with OTII CD4 T cells (green). Data recorded at 30-s intervals, over a period of 2 to 4 hours from the addition of T cells.

Movie S3. B–T_Conjugates_4.5–6.5hr.avi. *Dock8*$^{wt/wt}$ MD4 B cells (blue) and *Dock8*$^{cpm/cpm}$ MD4 B cells (magenta), activated overnight with 10 ng/ml of OVAHEL, forming conjugates with OTII CD4 T cells (green). Data recorded at 30-s intervals, over a period of 4.5 to 6.5 hours from the addition of T cells.

Movie S4. WT1_B+T+Actin_time-course.avi. *Dock8*$^{wt/wt}$ MD4 B cell (cyan), activated overnight with 10 ng/ml of OVAHEL and labeled with SiR-Actin (red), forming a conjugate with an OTII CD4 T cell (yellow). Data recorded for 20 min at 46-s intervals.

Movie S5. WT2_B+T+Actin_Z-stack.avi. *Dock8*$^{wt/wt}$ MD4 B cell (cyan), activated overnight with 10 ng/ml of OVAHEL and labeled with SiR-Actin (red), forming a conjugate with an OTII CD4 T cell (yellow). Data recorded for 30 min with 23 *z*-steps at 0.36 µm per step, recorded every 164 s.

Movie S6. CPM1_B+T+Actin_time-course.avi. *Dock8*$^{cpm/cpm}$ MD4 B cell (cyan), activated overnight with 10 ng/ml of OVAHEL and labeled with SiR-Actin (red), forming a conjugate with an OTII CD4 T cell (yellow). Data recorded for 20 min at 52-s intervals.

Movie S7. CPM2_B+T+Actin_Z-stack.avi. *Dock8*$^{cpm/cpm}$ MD4 B cell (cyan), activated overnight with 10 ng/ml of OVAHEL and labeled with SiR-Actin (red), forming a conjugate with an OTII CD4 T cell (yellow). Data recorded for 30 min with 20 *z*-steps at 0.36 µm per step, recorded every 164 s.

Movie S8. 3D-X-axis_WT2_B+T+Actin.avi. 3D view of a *Dock8*$^{wt/wt}$ MD4 B cell (cyan), activated overnight with 10 ng/ml of OVAHEL and labeled with SiR-Actin (red), forming a conjugate with an OTII CD4 T cell (yellow). 360-degree rotation along the *x*-axis from data recorded in movie S5.

Movie S9. 3D-Y-axis_WT2_B+T+Actin.avi. 3D view of a *Dock8*$^{wt/wt}$ MD4 B cell (cyan), activated overnight with 10 ng/ml of OVAHEL and labeled with SiR-Actin (red), forming a conjugate with an OTII CD4 T cell (yellow). 360-degree rotation along the *y*-axis from data recorded in movie S5.

Movie S10. 3D-X-axis_CPM2_B+T+Actin.avi. 3D view of a *Dock8*$^{cpm/cpm}$ MD4 B cell (cyan), activated overnight with 10 ng/ml of OVAHEL and labeled with SiR-Actin (red), forming a conjugate with an OTII CD4 T cell (yellow). 360-degree rotation along the *x*-axis from data recorded in movie S7.

Movie S11. 3D-Y-axis_CPM2_B+T+SiRActin.avi. 3D view of a *Dock8*$^{cpm/cpm}$ MD4 B cell (cyan), activated overnight with 10 ng/ml of OVAHEL and labeled with SiR-Actin (red), forming a conjugate with an OTII CD4 T cell (yellow). 360-degree rotation along the *y*-axis from data recorded in movie S7.

Data S1. (separate file)
List of top20 DEG that define 19 unique clusters at Res.0.9.

Data S2. (separate file)
List of DEG between *Dock8*$^{wt/wt}$ and *Dock8*$^{cpm/cpm}$ MD4 GC B cells.

Data S3. (separate file)
List of antibodies and other reagents used in the study.

Data S4. (separate file)
Tabulated data underlying main and supplementary figures.
